# Bioselectivity Induced by Chirality of New Terpenyl Organoselenium Compounds

**DOI:** 10.3390/ma12213579

**Published:** 2019-10-31

**Authors:** Magdalena Obieziurska, Agata J. Pacuła, Angelika Długosz-Pokorska, Marek Krzemiński, Anna Janecka, Jacek Ścianowski

**Affiliations:** 1 Department of Organic Chemistry, Faculty of Chemistry, Nicolaus Copernicus University, 7 Gagarin Street, 87-100 Torun, Poland; magdao@umk.pl (M.O.); pacula@umk.pl (A.J.P.); mkrzem@umk.pl (M.K.); 2 Department of Biomolecular Chemistry, Faculty of Medicine, Medical University of Lodz, Mazowiecka 6/8, 92-215 Lodz, Poland; angelika.dlugosz@umed.lodz.pl (A.D.-P.); anna.janecka@umed.lodz.pl (A.J.)

**Keywords:** benzisoselenazol-3(2H)-ones, terpenes, antioxidant activity, antiproliferative activity

## Abstract

A series of new chiral benzisoselenazol-3(2H)-ones substituted on the nitrogen atom with three monoterpene moieties—*p*-menthane, pinane and carane—was synthesized. The compounds were obtained by the reaction of 2-(chloroseleno)benzoyl chloride with an appropriate terpene amine, first synthesized by a multistep methodology starting from the corresponding alcohol (*p*-menthane system) or alkene (pinene and carene systems). Compounds were tested as antioxidants and anticancer agents. The *N*-isopinocampheyl-1,2-benzisoselenazol-3(2H)-one was the best peroxide scavenger and antiproliferative agent on the human promyelocytic leukemia cell line HL-60. The *N*-menthyl-1,2-benzisoselenazol-3(2H)-one revealed the highest anticancer potential towards breast cancer line MCF-7. The influence of structure and chirality on the bio-activity of the obtained organoselenium compounds was thoroughly evaluated.

## 1. Introduction

Organoselenium compounds are well-known for their remarkable redox properties and the ability to act as antioxidants, preventing oxidative stress, as well as pro-oxidants, able to inhibit proliferation of cancer cells [1,2]. Numerous in vitro and in vivo assays have proven that these compounds can be successfully applied as pharmacologically active agents [3]. However, finding bioavailable Se-molecules possessing high redox potential along with low toxicity is still the key issue in the field. Proper drug design can help to eliminate this problem and enable the construction of compounds that can selectively interact with specific targets in the human body [4]. Transformation of one compound to a different isomer, by changing the bonding arrangement (regioisomers) or the 3-dimensional orientation of atoms (epimers/enantiomers), can alter the biological activity of the molecule [5].

**Scheme 1:**
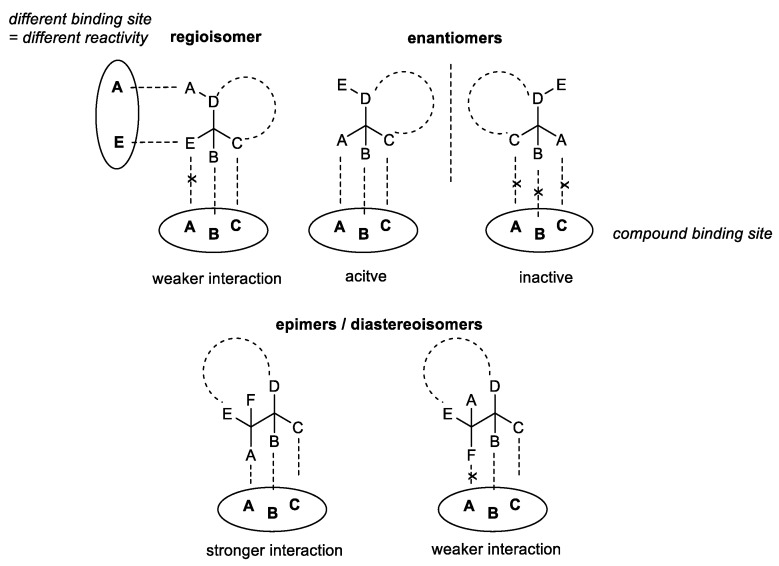
Interaction of different isomers with the target binding sites.

A chiral compound, possessing a strictly fixed orientation of substituents that fit to certain domains of the target, is able to exhibit a specific bio-capacity. On the contrary, its enantiomer, due to the opposite configuration of the asymmetric carbon center, is not able to interact. Additionally, if more than one chiral center is present in the molecule, we can observe two epimers/diastereoisomers with stronger or weaker drug-target interaction. The reactivity can also be modulated by transforming the compound to its regioisomer, possessing different atom organization but the same molecular formula. This way the strength of the binding can be changed or affinity for other domains can be developed (Scheme 1).

One molecular formula can be the source of various constitutional, conformational, or configurational isomers. Each can exhibit different biochemical and pharmacological properties, including transport, bioavailability, selectivity, metabolism, and excretion [6].

**Scheme 2:**
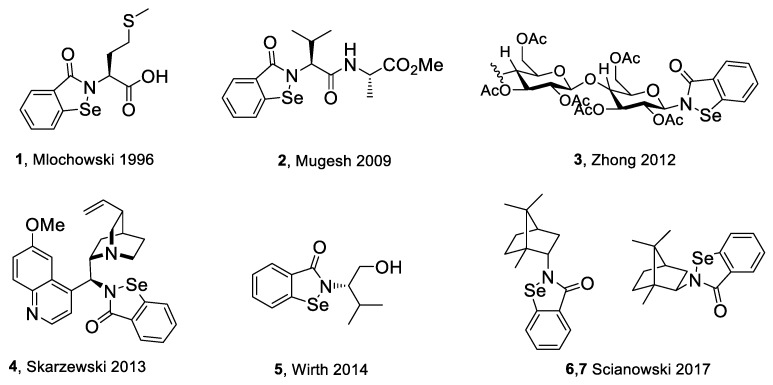
Structure of previously reported chiral benzisoselenazolones **1**–**7**.

The goal of this research was to synthetize new chiral benzisoselenazol-3(2H)-ones, functionalized on the nitrogen atom with monoterpene skeletons, test them as antioxidants and anticancer agents, and evaluate the particular structure–activity relationship. Although, there are some examples of chiral benzisoselenazolones, this group of compounds is still limited and the influence of specific chiral structures, including the difference between the activities of enantiomers, on the bio-capacity of the molecules has not yet been thoroughly evaluated. The known optically active benzisoselenazolones include, e.g., chiral alcohol **5 [7]** and also compounds of natural origin such as aminoacid **1** and **2** [8,9] monosaccharide **3** [10], and quinine **4** [11,12] derivatives, and reported in our research group, first *N*-terpenyl benzisoselenazolones **6**, **7** [13] (Scheme 2).

**Scheme 3:**
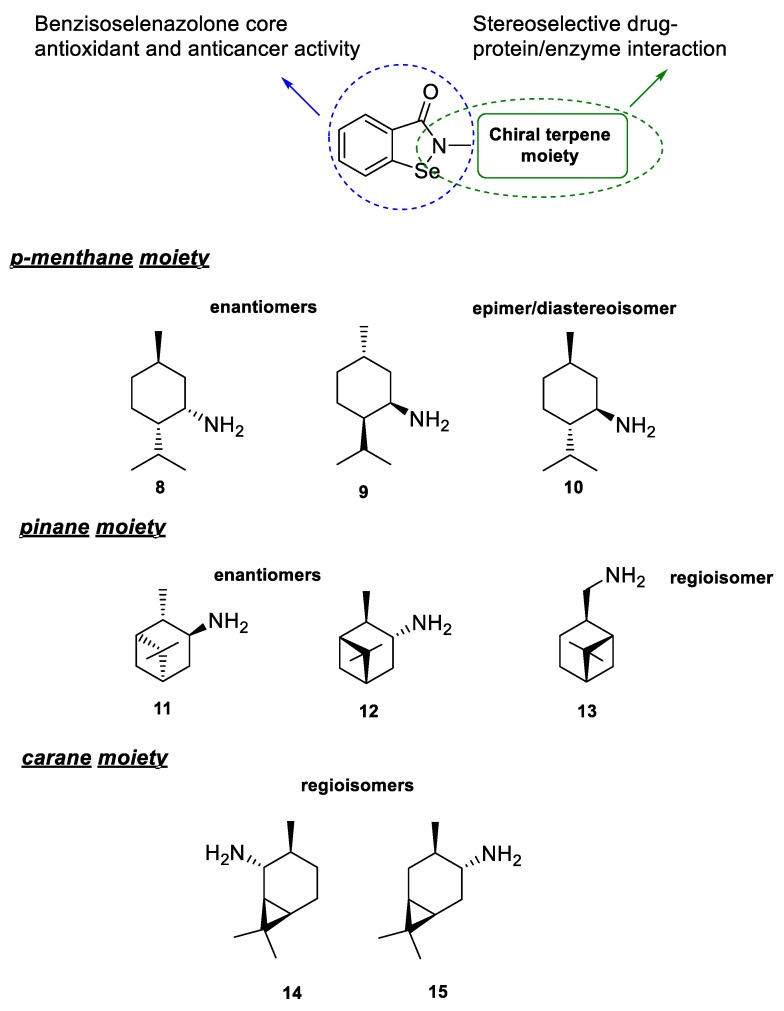
Structure of the designed benzisoselenazolones—activity correlation.

The presented research is based on the incorporation of a terpene moiety in the structure of antioxidant and anticancer organoselenium molecules. The designed compounds combine two main structural motifs. First, the benzisoselenazolone core, present in ebselen (*N*-phenylbenzisoselenazol-3(2H)-one), the most studied organoselenium compound with broad spectrum of possible therapeutic applications, that is responsible for, e.g., the GPx-like antioxidant activity [14]. Second, a chiral monoterpene moiety attached to the nitrogen atom that will be formed using optically active terpenyl amines **8**–**15**. The structures will include different enantiomers from the *p*-menthane **8**, **9** and pinane **11**, **12** systems, epimer of menthyl amine **10** and regioisomers **13**–**15** (Scheme 3).

The terpene amines **8**–**15**, selected for the functionalization of the benzisoselenazole core, include three types of previously presented (Scheme 1) isomeric forms—enantiomers, epimers, and regioisomers. The diversity of these structures enabled us to evaluate the correlation between the influence of the bulky aliphatic moiety and the 3-dimensional orientation of atoms on the bioactivity of all obtained compounds.

## 2. Materials and Methods

### 2.1. General

Melting points were measured with a Büchi Tottoli SPM-20 heating unit (Büchi Labortechnik AG, Flawil, Switzerland) and were uncorrected. NMR spectra were recorded on Bruker Avance III/400 or Bruker Avance III/700 (Karlsruhe, Germany) for 1H and 176.1 MHz or 100.6 MHz for 13C. Chemical shifts were recorded relative to SiMe4 (δ0.00) or solvent resonance (CDCl_3_ δ7.26, CD_3_OD δ3.31). Multiplicities were given as: s (singlet), d (doublet), dd (double doublet), ddd (double double doublet), t (triplet), dt (double triplet), and m (multiplet). 77Se NMR spectra were recorded on Bruker Avance III/ 400 or Bruker Avance III/ 700 with diphenyl diselenide as an external standard. NMR spectra were carried out using ACD/NMR Processor Academic Edition. Elemental analyses were performed on a Vario MACRO CHN analyzer. Optical rotations were measured in 10-mm cells with a polAAr 3000 polarimeter. Commercially available solvents DMF, DCM, and MeOH (Aldrich, St. Louis, MO, USA) and chemicals were used without further purification. Column chromatography was performed using Merck 40-63D 60Å silica gel (Merck, Darmstadt, Germany) and aluminium oxide.

### 2.2. Procedures and Analysis Data

#### 2.2.1. Synthesis of Caranyl Amines Hydrochlorides **14**, **15**

To a 250-mL two-necked round bottom flask, equipped with a magnetic stirring bar, septum, and inlet adapter connected to an argon line, 2-carene or 3-carene (210 mmol) and tetrahydrofuran (50 mL) were added. The flask was immersed in an ice-water bath and borane-methyl sulfide adduct (100 mmol) was added dropwise at 0–3 °C to the well-stirred reaction mixture. After addition, the stirring was terminated, the argon line removed and replaced with a rubber septum. The reaction flask was then placed in a 2–4 °C refrigerator for 24 h. After this time, the flask was connected to the argon line and supernatant was removed using a syringe with a long needle. The crystalline dialkylborane was washed with pentane (2 × 15 mL), washings were removed using a syringe and the solid was dried under vacuum (<1 mmHg) for 2 h. After this time, the flask was back-filled with argon and crystalline dialkylborane (**16**, **17**) was obtained with the yield in the range of 85–88%.

Tetrahydrofuran (50 mL) was added to diisocaranylboranes **16** or **17.** This suspension was cooled to 0 °C in an ice bath and 2-methylpropene (10 ml), which was condensed in a graduated cylinder immersed in a cooling bath (dry ice/acetone), was transferred to borane via double ended needle. Stirring was continued until a clear solution was obtained (about 2 h). Tetrahydrofuran (50 mL) was added, followed by the careful addition of hydroxylamine-O-sulfonic acid (15 g). After the addition was completed, the reaction mixture was heated under reflux for 5 h. The reaction mixture was cooled to room temperature and poured into a mixture of concentrated hydrochloric acid (80 mL) and ice (500 g). The water layer was extracted with diethyl ether (3 × 150 mL). The extracts were discarded and the acidic solution was treated with solid sodium hydroxide until pH of 10 was obtained. Then it was extracted with diethyl ether (3 × 100 mL). The combined extracts were dried with anhydrous magnesium sulfate, filtered, and evaporated on rotary evaporator under vacuum. The crude amine was dissolved in diethyl ether (100 mL) and hydrogen chloride solution in dioxane (4 M, 20 mL, 80 mmol) was added dropwise with stirring. Amine hydrochloride **14** or **15** was filtered off on Schott funnel and dried under vacuum with the yield in the range of 62–65%.

#### 2.2.2. Synthesis of Benzisoselenazol-3(2H)-Ones 30-37

To a solution of amine (2.0 mmol) and triethylamine (4.0 mmol) in dichloromethane (10 mL) 2-(chloroseleno)benzoyl chloride (2.0 mmol) was added. The mixture was stirred for 24 h at room temperature, poured on water and extracted with DCM. The combined organic layers were dried over anhydrous magnesium sulfate and evaporated. The crude product was purified by column chromatography (silica gel, chloroform-compounds **22**–**27**, **29**, aluminium oxide, chloroform-compound **28**).

(−)-*N*-(1*S*,2*S*,5*R*)-neomenthyl-1,2-benzisoselenazol-3(2H)-one **22** (Figure S1)

Yield: 78%; mp 130–132 °C;${\left[\propto \right]}_{D}^{20}$ = −41.49 (c = 0.94, CHCl_3_);

^1^H NMR (700 MHz, CDCl_3_) *δ* = 0.90 (d, *J* = 6.3 Hz, 3H, CH_3_), 0.91 (d, *J* = 6.3 Hz, 3H, CH_3_), 0.93 (d, *J* = 6.3 Hz, 3H, CH_3_), 1.35–1.38 (m, 1H), 1.43–1.48 (m, 2H), 1.56-1.61 (m, 2H), 1.92–1.97 (m, 3H), 2.02–2.05 (m, 1H), 5.39 (s, 1H), 7.45 (dt, *J* = 7.0, 1.2 Hz, 1H, 1H_ar_), 7.61 (dt, *J* = 7.0, 1.2 Hz, 1H, 1H_ar_), 7.64 (d, *J* = 7.7, 1H, 1H_ar_), 8.11 (d, *J* = 7.7, 1H, 1H_ar_) ppm; ^13^C NMR (100.6 MHz, CDCl_3_) *δ* = 20.94 (CH_3_), 21.21 (CH_3_), 22.51 (CH_3_), 26.88 (CH_2_), 27.22 (CH), 29.25 (CH), 34.58 (CH_2_), 40.91 (CH_2_), 46.59 (CH), 51.05 (CH), 123.01 (CH_ar_), 125.98 (CH_ar_), 126.81 (C_ar_), 128.85 (CH_ar_), 131.80 (CH_ar_), 138.61 (C_ar_), 167.64 (C=O) ppm; ^77^Se (76.3 MHz, CDCl_3_), *δ* = 899.58 ppm; IR: 2949, 2922, 2867, 1593, 1564, 1454, 1444, 1371, 1346, 1317, 1260, 1226, 1199, 1173, 1156, 1097, 1028 cm^−1^; Elemental Anal. Calcd. for C_17_H_23_NOSe (336.34): C, 60.71; H, 6.89; N, 4.16 Found: C, 60.93; H, 6.80; N, 4.02.

(+)-*N*-(1*R*,2*R*,5*S*)-neomenthyl-1,2-benzisoselenazol-3(2H)-one **23** (Figure S2)

Yield: 70%; mp 128–130 °C;${\left[\propto \right]}_{D}^{20}$ = +45.16 (c = 0.31, CHCl_3_);

^1^H NMR (400 MHz, CDCl_3_) *δ* = 0.88 (d, *J* = 6.3 Hz, 3H, CH_3_), 0.91 (d, *J* = 6.3 Hz, 3H, CH_3_), 0.93 (d, *J* = 6.3 Hz, 3H, CH_3_), 1.31–1.40 (m, 1H), 1.42–1.47 (m, 2H), 1.54–1.60 (m, 2H), 1.89–1.97 (m, 3H), 1.99–2.05 (m, 1H), 5.40 (s, 1H), 7.43 (dt, *J* = 7.0, 1.2 Hz, 1H, 1H_ar_), 7.60 (dt, *J* = 7.0, 1.2 Hz, 1H, 1H_ar_), 7.63 (d, *J* = 7.7, 1H, 1H_ar_), 8.10 (d, *J* = 7.7, 1H, 1H_ar_) ppm; ^13^C NMR (100.6 MHz, CDCl_3_) *δ* = 20.97 (CH_3_), 21.22 (CH_3_), 22.53 (CH_3_), 26.87 (CH_2_), 27.18 (CH), 29.23 (CH), 34.57 (CH_2_), 40.91 (CH_2_), 46.57 (CH), 51.01 (CH), 123.16 (CH_ar_), 125.95 (CH_ar_), 126.82 (C_ar_), 128.79 (CH_ar_), 131.78 (CH_ar_), 138.72 (C_ar_), 167.67 (C=O) ppm; ^77^Se (76.3 MHz, CDCl_3_), *δ* = 899.30 ppm; IR: 2949, 2922, 2867, 1593, 1564, 1454, 1444, 1371, 1346, 1317, 1260, 1226, 1199, 1173, 1156, 1097, 1028 cm^−1^; Elemental Anal. Calcd. for C_17_H_23_NOSe (336.34): C, 60.71; H, 6.89; N, 4.16 Found: C, 60.48; H, 6.98; N, 4.19.

(−)-*N*-(1*R*,2*S*,5*R*)-menthyl-1,2-benzizoselenazol-3(2H)-one **24** (Figure S3)

Yield: 86%, mp 188–190 °C; ${\left[\propto \right]}_{D}^{20}$ = −53.76 (c = 1.86, CHCl_3_);

^1^H NMR (400 MHz, CDCl_3_) *δ* = 0.83 (d, *J* = 6.8 Hz, 3H, CH_3_), 0.90 (d, *J* = 7.2 Hz, 3H, CH_3_), 0.95 (d, *J* = 6.4 Hz, 3H, CH_3_), 1.13–1.28 (m, 4H), 1.62–1.71 (m, 1H), 1.74–1.81 (m, 3H), 2.01–2.08 (m, 1H), 4.54 (s, 1H), 7.43 (dt, *J* = 7.6, 1.2 Hz, 1H, 1H_ar_), 7.58 (dt, *J* = 7.2, 1.6 Hz, 1H, 1H_ar_), 7.64 (d, *J* = 7.6, 1H, 1H_ar_), 8.06 (d, *J* = 7.2, 1H, 1H_ar_) ppm; ^13^C NMR (100.6 MHz, CDCl_3_) *δ* = 15.87 (CH_3_), 21.25 (CH_3_), 22.10 (CH_3_), 23.76 (CH_2_), 26.39 (CH), 32.18 (CH), 34.40 (CH_2_), 43.61 (CH_2_), 49.55 (CH), 54.75 (CH), 123.95 (CH_ar_), 126.04 (CH_ar_), 128.42 (C_ar_), 128.82 (CH_ar_), 131.63 (CH_ar_), 137.79 (C_ar_), 166.86 (C=O) ppm; ^77^Se (76.3 MHz, CDCl_3_), *δ* = 828.67 ppm; IR: 2955, 2924, 2870, 2849, 1590, 1561, 1459, 1442, 1370, 1352, 1327, 1310, 1285, 1270, 1248, 1185, 1143, 1053, 1026, 1001 cm^−1^; Elemental Anal. Calcd. for C_17_H_23_NOSe (336.34): C, 60.71; H, 6.89; N, 4.16 Found: C, 61.27; H, 6.73, N, 4.08.

(+)-*N*-(1*S*,2*S*,3*S*,5*R*)-isopinocampheyl-1,2-benzisoselenazol-3(2H)-one **25** (Figure S4)

Yield: 48%; mp 135–138 °C; ${\left[\propto \right]}_{D}^{20}$ = +17.52 (c = 0.75, CHCl_3_);

^1^H NMR (700 MHz, CDCl_3_) 1.07 (d, *J* = 10.5 Hz, 1H), 1.16 (s, 3H, CH_3_), 1.17 (d, *J* = 7.0 Hz, 3H, CH_3_), 1.27 (s, 3H, CH_3_), 1.83-1.86 (m, 1H), 1.91–1.93 (m, 1H), 2.03–2.06 (m, 1H), 2.15–2.20 (m, 1H), 2.51–2.55 (m, 1H), 2.61–2.65 (m, 1H), 5.13–5.16 (m, 1H), 7.42 (dt, *J* = 7.7, 0.7 Hz, 1H, 1H_ar_), 7.56 (dt, *J* = 7.7, 1.4 Hz, 1H, 1H_ar_), 7.63 (d, *J* = 7.0, 1H, 1H_ar_), 8.02 (d, *J* = 7.0, 1H, 1H_ar_) ppm; ^13^C NMR (176.1 MHz, CDCl_3_) *δ* = 20.04 (CH_3_), 23.13 (CH_3_), 27.60 (CH_3_), 35.60 (CH_2_), 35.78 (CH_2_), 37.65 (C), 41.43 (CH), 46.04 (CH), 47.65 (CH), 52.49 (CH), 123.45 (CH_ar_), 125.80 (CH_ar_), 128.23 (C_ar_), 128.52 (CH_ar_), 131.30 (CH_ar_), 137.15 (C_ar_), 166.90 (C = O) ppm; ^77^Se (76.3 MHz, CDCl_3_), *δ* = 804.13 ppm; IR: 2924, 2871, 1608, 1585, 1561, 1453, 1440, 1372, 1339, 1322, 1308, 1284, 1246, 1225, 1182, 1156, 1058, 1045, 1019, 1004 cm^−1^; Elemental Anal. Calcd. for C_17_H_21_NOSe (334.32): C, 61.08; H, 6.33; N, 4.19 Found: C, 61.47; H, 6.43; N, 4.02.

(−)-*N*-(1*R*,2*R*,3*R*,5*S*)-isopinocampheyl-1,2-benzisoselenazol-3(2H)-one **26** (Figure S5)

Yield: 59%; mp 138–140 °C; ${\left[\propto \right]}_{D}^{20}$ = −17.32 (c = 0.85, CHCl_3_)

^1^H NMR (400 MHz, CDCl_3_) 1.08 (d, *J* = 10.5 Hz, 1H), 1.17 (s, 3H, CH_3_), 1.18 (d, *J* = 7.0 Hz, 3H, CH_3_), 1.28 (s, 3H, CH_3_), 1.83–1.88 (m, 1H), 1.92–1.96 (m, 1H), 2.04–2.08 (m, 1H), 2.16–2.22 (m, 1H), 2.51–2.57 (m, 1H), 2.61–2.66 (m, 1H), 5.13–5.19 (m, 1H), 7.43 (dt, *J* = 7.7, 0.7 Hz, 1H, 1H_ar_), 7.57 (dt, *J* = 7.7, 1.4 Hz, 1H, 1H_ar_), 7.65 (d, *J* = 7.0, 1H, 1H_ar_), 8.03 (d, *J*= 7.0, 1H, 1H_ar_) ppm; ^13^C NMR (176.1 MHz, CDCl_3_) *δ* = 21.44 (CH_3_), 24.48 (CH_3_), 29.00 (CH_3_), 36.98 (CH_2_), 37.18 (CH_2_), 39.03 (C), 42.85 (CH), 47.39 (CH), 49.08 (CH), 53.90 (CH), 124.97 (CH_ar_), 127.14 (CH_ar_), 129.66 (C_ar_), 129.84 (CH_ar_), 132.65 (CH_ar_), 138.68 (C_ar_), 168.31 (C=O) ppm; ^77^Se (76.3 MHz, CDCl_3_), *δ* = 805.29 ppm; IR: 2924, 2871, 1608, 1585, 1561, 1453, 1440, 1372, 1339, 1322, 1308, 1284, 1246, 1225, 1182, 1156, 1058, 1045, 1019, 1004 cm^−1^; Elemental Anal. Calcd. for C_17_H_21_NOSe (334.32): C, 61.08; H, 6.33; N, 4.19 Found: C, 61.39; H, 6.24; N, 4.23.

(−)-*N*-(1*S*,2*R*,5*S*)-myrtanyl-1,2-benzisoselenazol-3(2H)-one **27** (Figure S6)

Yield: 65%; mp 133–135 °C; ${\left[\propto \right]}_{D}^{20}$ = −66.67 (c = 0.84, CHCl_3_) 

^1^H NMR (700 MHz, CDCl_3_) 0.92 (d, *J* = 9.8 Hz, 1H), 1.17 (s, 3H, CH_3_), 1.21 (s, 3H, CH_3_), 1.57–1.63 (m, 1H), 1.84–1.89 (m, 1H), 1.91–2.01 (m, 4H), 2.34–2.38 (m, 1H), 2.46–2.51 (m, 1H), 3.77–3.80 (m, 1H), 3.95–3.98 (m, 1H), 7.42 (dt, *J* = 7.0, 1.4 Hz, 1H, 1H_ar_), 7.58 (dt, *J* = 7.7, 0.7 Hz, 1H, 1H_ar_), 7.61 (d, *J* = 7.7, 1H, 1H_ar_), 8.04 (d, *J* = 7.7, 1H, 1H_ar_) ppm; ^13^C NMR (100.6 MHz, CDCl_3_) *δ* = 19.40 (CH_2_), 23.24 (CH_3_), 25.92 (CH_2_), 27.84 (CH_3_), 32.99 (CH_2_), 38.74 (C), 41.31 (CH), 42.37 (CH), 43.51 (CH), 50.28 (CH_2_), 123.90 (CH_ar_), 126.16 (CH_ar_), 127.63 (C_ar_), 128.88 (CH_ar_), 131.84 (CH_ar_), 137.78 (C_ar_), 167.31 (C=O) ppm; ^77^Se (76.3 MHz, CDCl_3_), *δ* = 889.73 ppm; IR: 2913, 2863, 1593, 1558, 1468, 1455, 1441, 1372, 1343, 1324, 1307, 1245, 1223, 1187, 1156, 1018 cm^−1^; Elemental Anal. Calcd. for C_17_H_21_NOSe (334.32): C, 61.08; H, 6.33; N, 4.19 Found: C, 61.36; H, 6.29; N, 4.09.

(−)-*N*-(1*S*,2*R*,3*S*,6*R*)-(2-isocaranyl)-1,2-benzisoselenazol-3(2H)-one **28** (Figure S7)

Yield: 57%; mp 139–142 °C; ${\left[\propto \right]}_{D}^{20}$ = −24.69 (c = 0.81, CHCl_3_)

^1^H NMR (700 MHz, CDCl_3_) 0.70–0.74 (m, 1H), 0.85 (d, *J* = 6.3 Hz, 3H, CH_3_), 0.99 (s, 3H, CH_3_), 1.02–1.05 (m, 1H), 1.16 (s, 3H, CH_3_), 1.41–1.46 (m, 1H), 1.61–1.65 (m, 1H), 1.75–1.78 (m, 1H), 1.83–1.89 (m, 1H), 4.14–4.16 (m, 1H), 7.42 (dt, *J* = 7.7, 0.7 Hz, 1H, 1H_ar_), 7.57 (dt, *J* = 8.4, 1.4 Hz, 1H, 1H_ar_), 7.65 (d, *J* = 8.4, 1H, 1H_ar_), 8.05 (d, *J* = 7.7, 1H, 1H_ar_) ppm;^13^C NMR (100.61 MHz, CDCl_3_) *δ* = 15.90 (CH_3_), 18.08 (C), 18.55 (CH_3_), 19.06 (CH_2_), 20.71 (CH), 28.58 (CH_3_), 29.10 (CH_3_), 30.93 (CH_2_), 36.13 (CH), 54.60 (CH), 124.03 (CH_ar_), 126.10 (CH_ar_), 128.27 (C_ar_), 128.88 (CH_ar_), 131.68 (CH_ar_), 137.94 (C_ar_), 167.27 (C=O) ppm; ^77^Se (76.3 MHz, CDCl_3_), *δ* = 826.55 ppm; IR: 2923, 2862, 1612, 1568, 1443, 1375, 1365, 1335, 1303, 1253, 1180, 1114, 1061, 1040, 1020 cm^−1^; Elemental Anal. Calcd. for C_17_H_21_NOSe (334.32): C, 61.08; H, 6.33; N, 4.19 Found: C, 61.01; H, 6.31; N, 4.05.

(−)-*N*-(1*S*,3*R*,4*R*,6*R*)-(4-isocaranyl)-1,2-benzisoselenazol-3(2H)-one **29** (Figure S8)

Yield: 57%; mp 62–54 °C; ${\left[\propto \right]}_{D}^{20}$ = −40.00 (c = 1.15, CHCl_3_)

^1^H NMR (700 MHz, CDCl_3_) 0.73–0.75 (m, 1H), 0.77–0.80 (m, 1H), 0.81 (d, *J* = 7.0 Hz, 3H, CH_3_), 0.91–0.99 (m, 1H), 1.01 (s, 3H, CH_3_), 1.08 (s, 3H, CH_3_), 1.39–1.46 (m, 1H), 1.78–1.82 (m, 1H), 2.09–2.13 (m, 1H), 2.15–2.17 (m, 1H), 4.13–4.17 (m, 1H), 7.41 (dt, *J* = 7.7, 1.4 Hz, 1H, 1H_ar_), 7.56 (dt, *J* = 7.7, 1.4 Hz, 1H, 1H_ar_), 7.63 (d, *J* = 7.7, 1H, 1H_ar_), 8.01 (d, *J* = 7.7, 1H, 1H_ar_) ppm; ^13^C NMR (100.61 MHz, CDCl_3_) *δ* = 15.88 (CH_3_), 18.04 (C), 18.16 (CH_3_), 20.24 (CH), 20.96 (CH), 28.48 (CH_2_), 28.83 (CH_3_), 29.11 (CH_2_), 35.63 (CH), 56.99 (CH), 123.93 (CH_ar_), 126.09 (CH_ar_), 128.37 (C_ar_), 128.81 (CH_ar_), 131.71 (CH_ar_), 137.63 (C_ar_), 167.48 (C=O) ppm; ^77^Se (76.3 MHz, CDCl_3_), *δ* = 818.75 ppm; IR: 2925, 2860, 1589, 1563, 1443, 1365, 1337, 1307, 1243, 1182, 1057, 1043, 1020 cm^−1^; Elemental Anal. Calcd. for C_17_H_21_NOSe (334.32): C, 61.08; H, 6.33; N, 4.19 Found: C, 61.23; H, 6.40; N, 4.25.

### 2.3. Antioxidant Activity Assay

To a solution of compounds **30**–**37** (0.015mmol) and dithiothreitol DTT^red^ (0.15mmol) in 1.1 mL of CD_3_OD, 30% H_2_O_2_ (0.15 mmol) was added. ^1^H NMR spectra were measured right after addition of hydrogen peroxide and then in the specific time intervals. The concentration of the substrate was determined according to the changes in the integration on the ^1^H NMR spectra [15].

### 2.4. MTT Viability Assay 

The MTT assay was based on the method of Mosmann [16].

## 3. Results and Discussion

The first step of the research involved the synthesis of chiral amines from three monoterpene systems: *p*-menthane, pinane, and carane. The compounds were obtained by two different procedures. In the case of *p*-menthane derived amines **8**–**9**, they were obtained by known methodology based on the reduction of corresponding oximes [17]. Enantiomer **10** was commercially available. Pinene-derived amines **11**–**13** were obtained by the two-step procedure from (-)-α-pinene, (+)-α-pinene, and (-)-β-pinene. The appropriate alkene was hydroborated to boranes, which were than cleaved with hydroxylamine-O-sulfonic acid and acidified to form 3-pinanylamine hydrochlorides **11**, **12** and the regioisomeric 10-pinanylamine hydrochloride **13** [18,19].

**Scheme 4:**
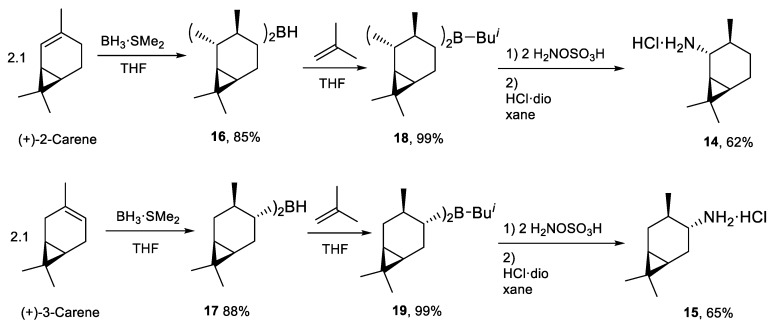
Synthesis of amines derived from the carane group.

Other bicyclic amines **14**, **15** [19] were synthesized from (+)-2-carene and (+)-3-carene. The procedure was a modification of the one previously used for the pinane system [18]. To increase the yield of the final product, diisocaranylboranes **16** and **17** reacted with 2-methylpropene to form trialkylboranes. This newly proposed method enabled to obtain diisocaranylisobuthylboranes **18** and **19**, which were efficiently transformed to amines **14** and **15** (Scheme 4).

**Scheme 5:**
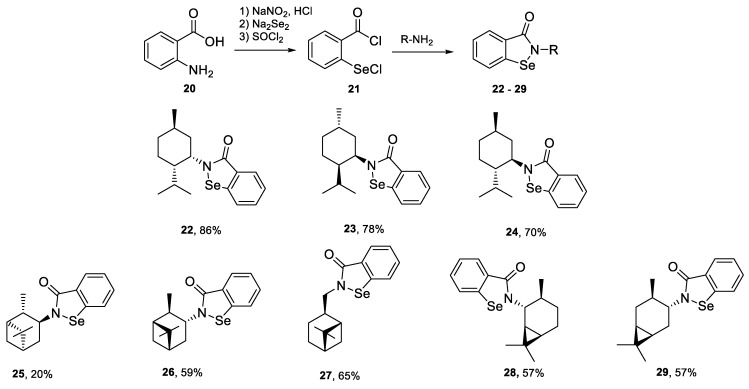
Synthesis of *N*-terpenyl benzisoselenazol-3(2H)-ones **30**–**37**.

All synthesized amines were further transformed to benzisoselenazol-3(2H)-ones **22**–**29**. We have applied our previously reported method based on the reaction of 2-(chloroseleno)benzoyl chloride **21**, obtained by a multistep procedure starting from anthranilic acid **20**, with an appropriate amine to form the final organoselenium derivatives **22**–**29** (Scheme 5) [13].

**Figure 1:**
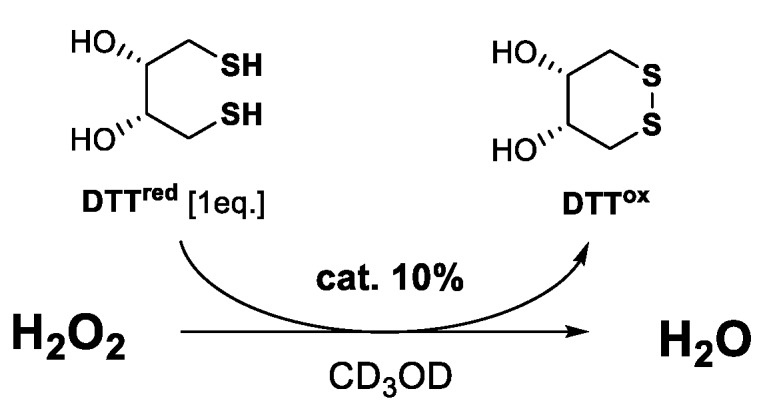
Antioxidant activity test.

Finally, the antioxidant and antiproliferative activities of benzisoselenazolones **22**–**29** were evaluated. As presented in Figure 1, first, the ability to reduce H_2_O_2_ was tested by a frequently used procedure, proposed by Iwaoka and co-workers, where the Se-catalyst reduces the peroxide and, in the oxidized form, is able to transform the dithiol (DTT^red^) to a disulfide (DTT^ox^). The rate of the reaction is equal to the conversion of –SH to –S–S– observed on ^1^H NMR spectra in the specific time intervals (Table 1) [15].

The most efficient H_2_O_2_ reduction was found for the pinene derivatives **25** and **27**, with the total substrate conversion observed in 30 and 60 min, respectively. This result correlates with our previous investigations, in which also the bulky bicyclic bornane skeletons, in the structure of compounds **6** and **7** (Scheme 2), exhibited the highest antioxidant potential with total DTT^red^ oxidation time of 5 minutes.

Next, the antiproliferative capacity was measured by the cell viability assay (MTT) on breast cancer MCF-7 and human promyelocytic leukemia HL-60 cell lines [16]. The results are presented in Table 2.

The *N*-isopinocampheyl-1,2-benzisoselenazol-3(2H)-one **25** exhibited the highest antiproliferative potential with IC_50_ of 7.1 ± 0.4 µM (HL-60 cell line). In the case of all derivatives from the pinane system, the enantiomer **25** was the most active on both cell lines. Attachment of the isoselenazolone ring to the C10 carbon decreased the activity, what was highly emphasized in the results obtained for HL-60 cells with the increase of IC_50_ from 7.1 ± 0.4 to 250 ± 24.7 µM.

**Scheme 6:**
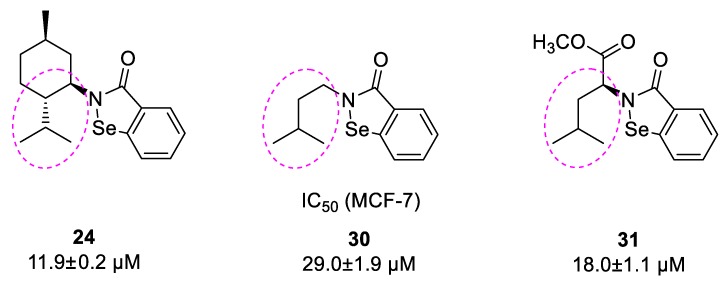
Repetitive carbon chain in the structure of bio-active derivatives **24**, **30**, and **31**.

The best anticancer activity against MCF-7 cells was observed for *N*-menthyl-1,2-benzisoselenazol-3(2H)-one **24** with IC_50_ of 11.9 ± 0.2 µM. Moreover, the molecule possesses the same structural motif that was present in the structure of other active *N*-alkyl benzisoselenazolones **30** and **31**—an incorporated 2-methylbutyl chain (Scheme 6).

**Scheme 7:**
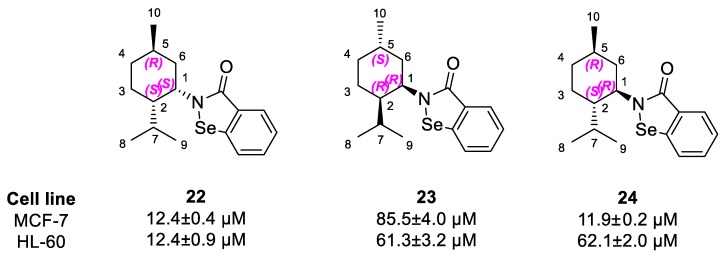
Similarity in the stereochemistry of derivatives **30**–**32**.

As we have reported before, this carbon chain could be a pharmacophore essential for the molecule to interact with the biological target [13,20]. Interestingly, we have also observed a different activity for both enantiomeric pairs derived from *p*-menthane and pinane. The diversity was more apparent in the case of compounds **22** and **23**, where one enantiomer was far more active with IC_50_ values 12.4 ± 0.4 µM and 85.5 ± 4.0 µM, respectively. Compounds **22** and **24**, with good antiproliferative capacity, have the same configurations on C1 and C4 carbon atoms, as opposed to the less active enantiomer **23**. The stereochemistry of C2, attached to the isoselenazolone ring, seems not to influence the activity (Scheme 7).

In the case of carane regioisomers **28** and **29** the IC_50_ was lower when the N-C_terpene_ bond was more hindered by the methyl group and the cyclopropane bridge. The overall activity decreased when the compounds were tested on HL-60 cell line.

## 4. Conclusions

In this paper we have presented the synthesis of the series of *N*-terpenyl benzisoselenazol-3(2H)-ones derived from three monoterpene systems: *p*-menthane, pinane and carane. The procedure was based on the reaction of 2-(chloroseleno)benzoyl chloride with an appropriate terpene monocyclic or bicyclic amine. The obtained eight chiral organoselenium compounds included enantiomers, epimers, and regioisomers were further tested as potential antioxidants and anticancer agents with structure–activity evaluation. The best antioxidant activity was observed for *α*- and *β*-pinane *N*-substituted benzisoselenazolones. The highest antiproliferative potential was observed for derivatives with α-pinane and *p*-menthane skeletons. This was in good agreement with our previous research, where the presence of an incorporated 2-methylbutyl chain increased the anticancer activity. Taking into account the significant difference in the reactivity of enantiomers: (-)-*N*-neomenthyl- and (+)-*N*-neomenthylbenzisoselenazol-3(2H)-one, it was also assumed that the configuration at C1 and C4 carbons can be essential for the improvement of the bio-activity. The results obtained for pinene and carene derivatives indicate that the more bulky the terpene system and the more hindered the *N*-C_terpene_ bond the higher the anticancer potential.

## Figures and Tables

**Table 1:** Results of the antioxidant activity measurement.

	Remaining DTT^red^ [%]				
Catalyst [0.1 equiv.]	3 min	5 min	15 min	30 min	60 min
**Benzisoselenazolones**					
**22**	91	85	75	63	49
**24**	80	77	74	70	67
**25**	**71**	**39**	**5**	**0**	**0**
**27**	**74**	**61**	**28**	**6**	**0**
**28**	92	89	83	74	58
**29**	69	61	43	40	39
**Ebselen**	84	75	64	58	52

**Table 2:** Results of the antiproliferative activity measurement.

Compound	Structure	IC_50_ [µM]	
		MCF-7	HL-60
**22**	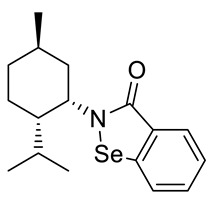	12.4 ± 0.4	12.4 ± 0.9
**23**	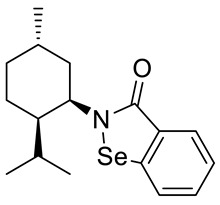	85.5 ± 4.0	61.3 ± 3.2
**24**	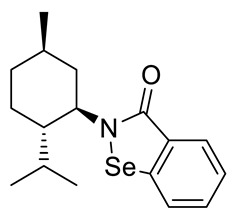	11.9 ± 0.2	62.1 ± 2.0
**25**	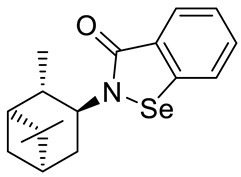	19.9 ± 0.4	7.1 ± 0.4
**26**	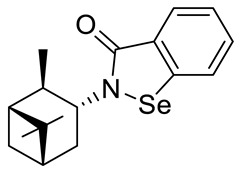	13.3 ± 1.1	20.6 ± 1.0
**27**	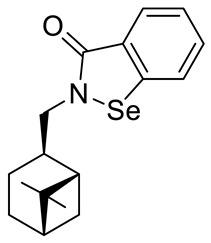	45.3 ± 2.1	250 ± 24.7
**28**	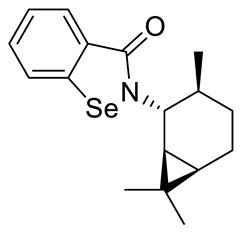	24.3 ± 2.4	203 ± 2.0
**29**	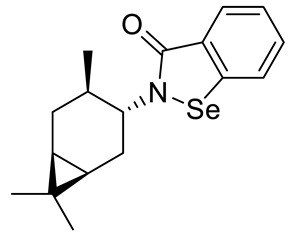	45.2 ± 2.1	210 ± 14.1

## Supplementary Materials

Supplementary materials are available online: https://www.mdpi.com/1996-1944/12/21/3579/s1, Figure S1: (a) ^1^H NMR, (b) ^13^C NMR, and (c) ^77^Se NMR spectra of (–)-*N*-(1*S*,2*S*,5*R*)-neomenthyl-1,2-benzisoselenazol-3(2H)-one **22**. Figure S2: (a) ^1^H NMR, (b) ^13^C NMR, and (c) ^77^Se NMR spectra of (+)-*N*-(1*R*,2*R*,5*S*)-neomenthyl-1,2-benzisoselenazol-3(2H)-one **23**. Figure S3: (a) ^1^H NMR, (b) ^13^C NMR, and (c) ^77^Se NMR spectra of (−)-*N*-(1*R*,2*S*,5*R*)-menthyl-1,2-benzizoselenazol-3(2H)-one **24**. Figure S4: (a) ^1^H NMR, (b) ^13^C NMR, and (c) ^77^Se NMR spectra (−)-*N*-(1*S*,2*S*,3*S*,5*R*)-isopinocampheyl-1,2-benzisoselenazol-3(2H)-one **25**. Figure S5: (a) ^1^H NMR, (b) ^13^C NMR, and (c) ^77^Se NMR spectra (+)-*N*-(1*R*,2*R*,3*R*,5*S*)-isopinocampheyl-1,2-benzisoselenazol-3(2H)-one **26**. Figure S6: (a) ^1^H NMR, (b) ^13^C NMR, and (c) ^77^Se NMR spectra of (−)-*N*-(1*S*,2*R*,5*S*)-myrtanyl-1,2-benzisoselenazol-3(2H)-one **27**. Figure S7: (a) ^1^H NMR, (b) ^13^C NMR, and (c) ^77^Se NMR spectra of (−)-*N*-(1*S*,2*R*,3*S*,6*R*)-(2-isocaranyl)-1,2-benzisoselenazol-3(2H)-one **28**. Figure S8: (a) ^1^H NMR, (b) ^13^C NMR, and (c) ^77^Se NMR −)-*N*-(1*S*,3*R*,4*R*,6*R*)-(4-isocaranyl)-1,2-benzisoselenazol-3(2H)-one **29**.
