# Supplementary material for: Bioselectivity Induced by Chirality of New Terpenyl Organoselenium Compounds
Source: Materials (Basel). 2019 Oct 31;12(21):3579. doi: 10.3390/ma12213579 (PMC6862013; doi:10.3390/ma12213579)
Supplement: Supplementary file 1 [file materials-12-03579-s001.pdf]

materials

MDPI

Supplementary Materials

Bioselectivity Induced by Chirality of New Terpenyl Organoselenium Compounds

Magdalena Obieziurska [1], Agata J. Pacuła [1], Angelika Długosz-Pokorska [2], Marek Krzemiński [1,†], Anna Janecka [2] and Jacek Ścianowski [1,*]

[1] Department of Organic Chemistry, Faculty of Chemistry, Nicolaus Copernicus University, 7 Gagarin Street, 87-100 Torun, Poland; magdao@umk.pl (M.O.); pacula@umk.pl (A.J.P.); mkrzem@umk.pl (M.K.)

[2] Department of Biomolecular Chemistry, Faculty of Medicine, Medical University of Lodz, Mazowiecka 6/8, 92-215 Lodz, Poland; angelika.dlugosz@umed.lodz.pl (A.D.-P.); anna.janecka@umed.lodz.pl (A.J.)

* Correspondence: jsch@umk.pl

† Dedicated to Professor Marek Zaidlewicz on the occasion of his 80th birthday

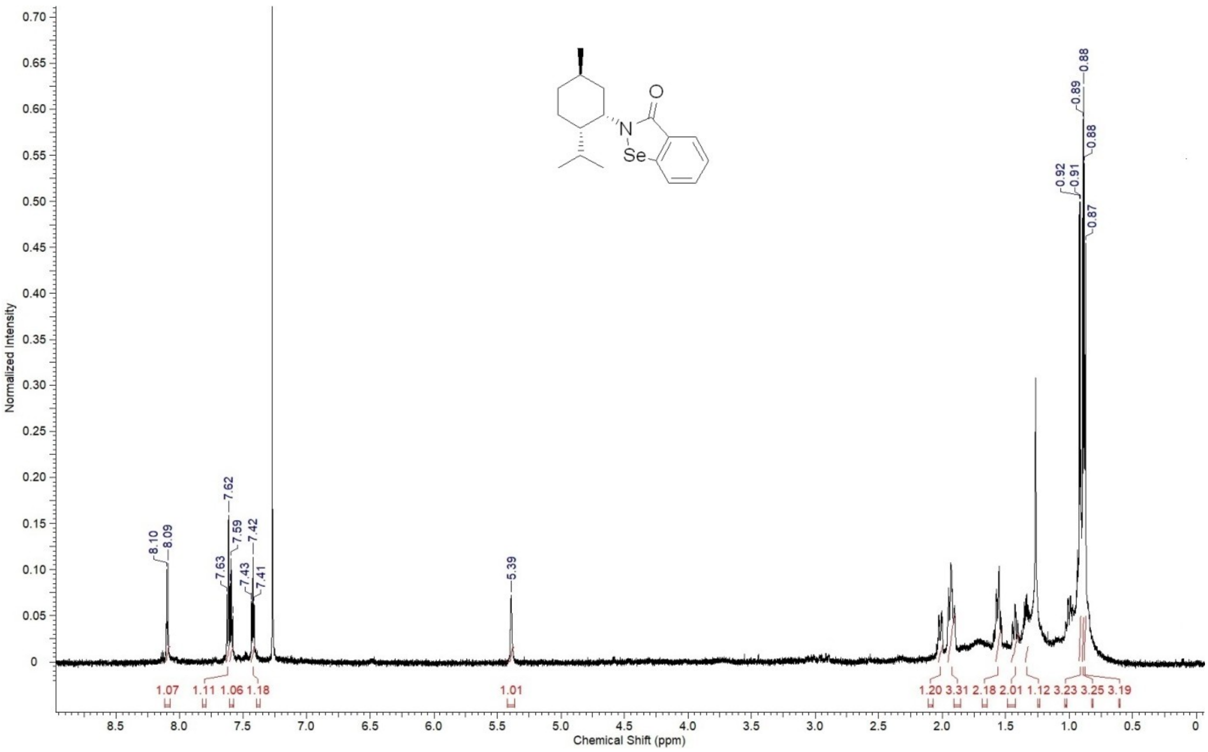

(a)

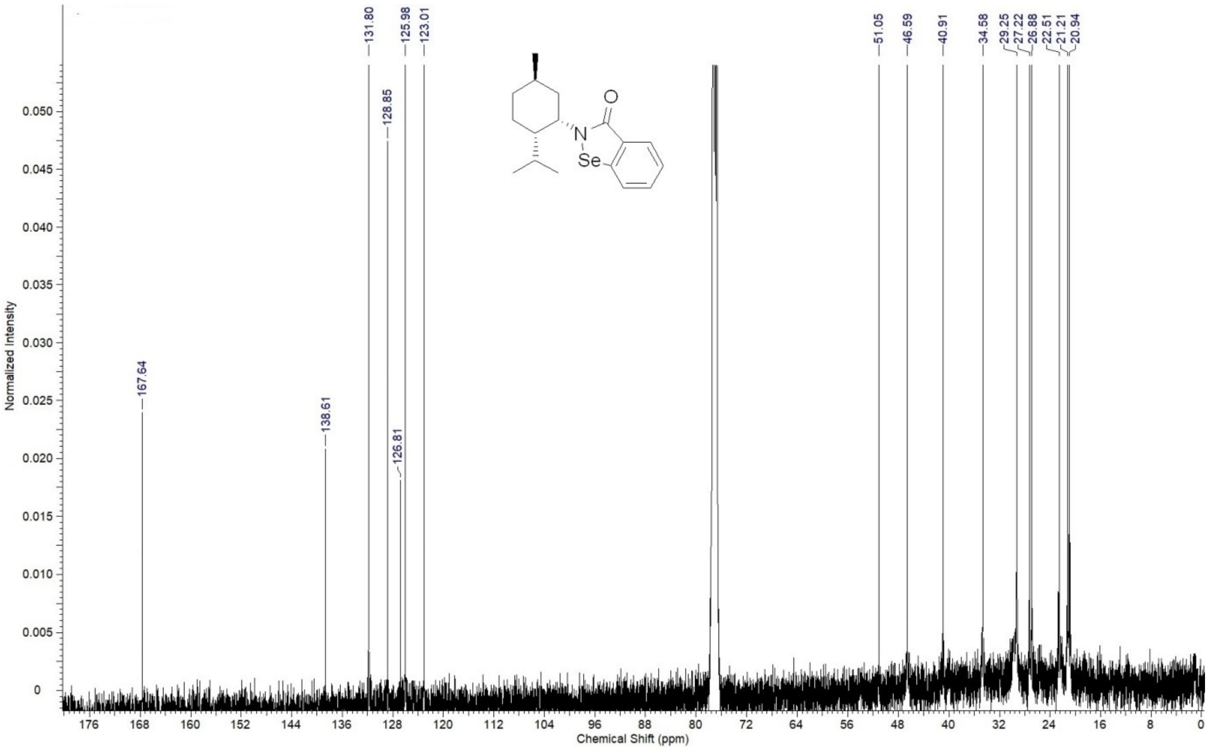

(b)

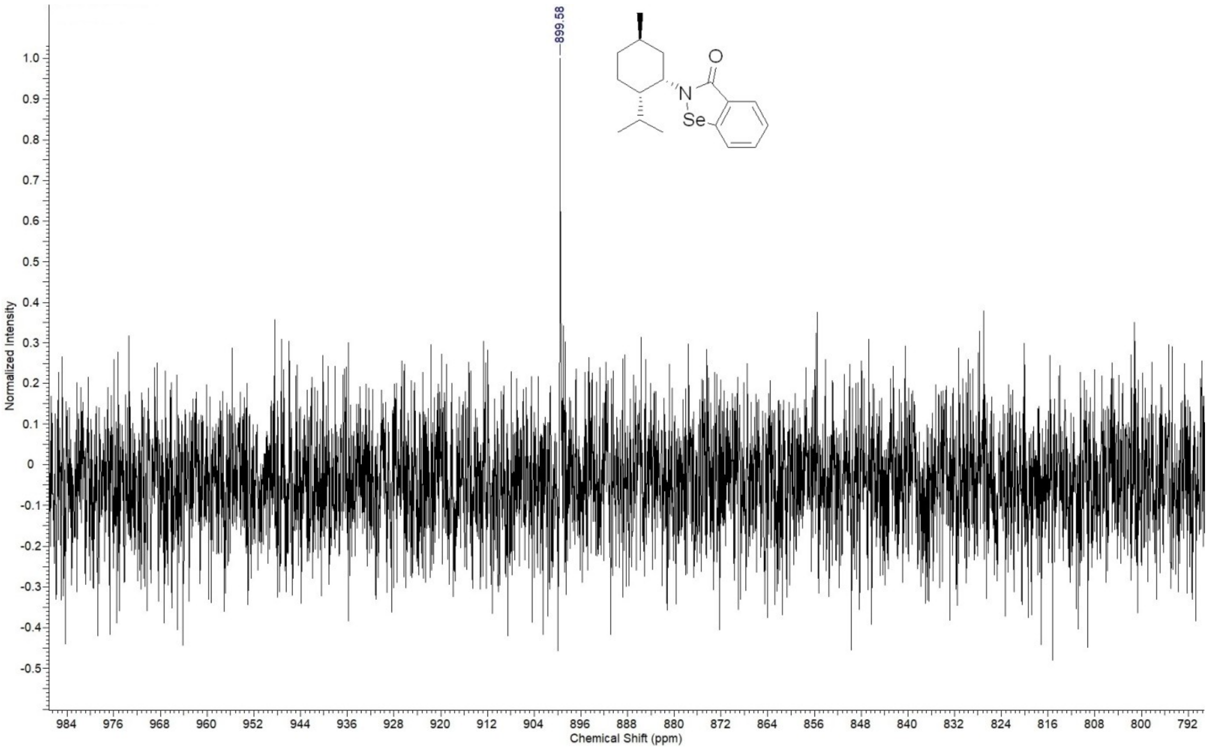

(c)

Figure S1. (a) ^{1}H NMR, (b) ^{13}C NMR, and (c) ^{77}Se NMR spectra of (–)-*N*-(1*S*,2*S*,5*R*)-neomenthyl-1,2-benzisoselenazol-3(2H)-one **22.**

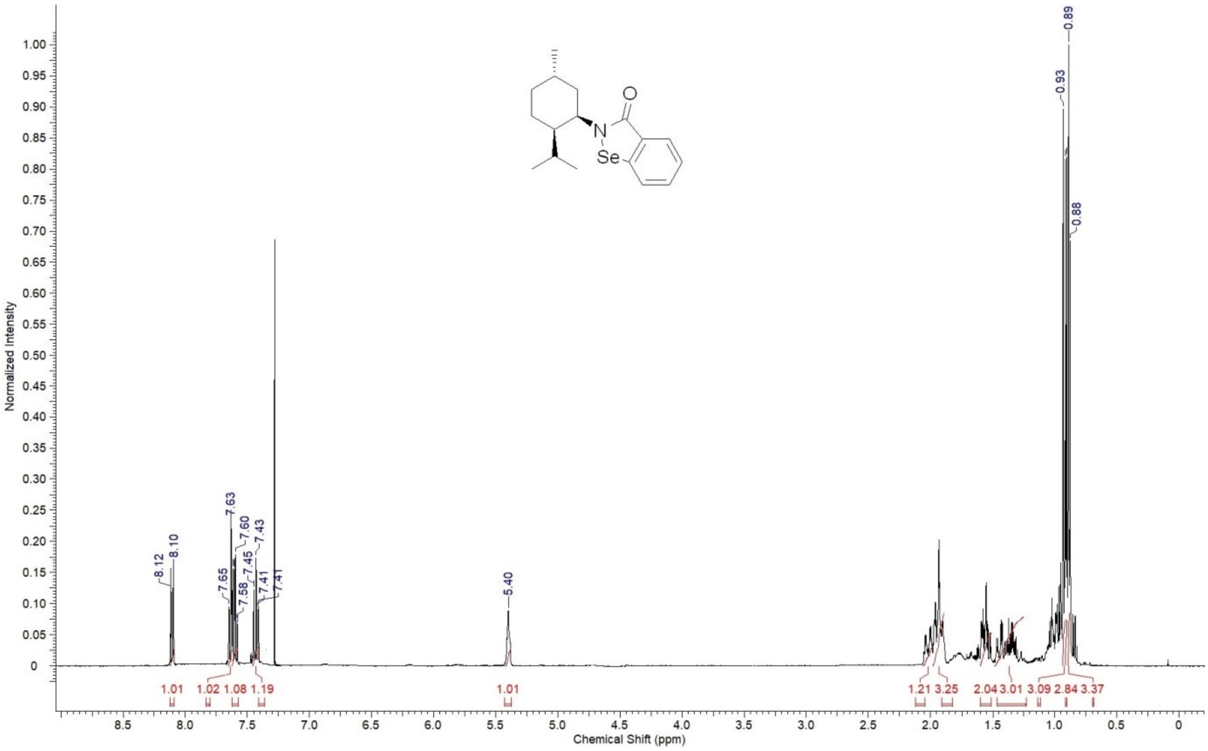

Normalized Intensity
Chemical Shift (ppm)
8.12 8.10 7.65 7.63 7.60 7.58 7.45 7.43 7.41 7.41 5.40 0.93 0.89 0.88
1.01 1.02 1.08 1.19 1.01 1.21 3.25 2.04 3.01 3.09 2.84 3.37
N
Se
O

(a)

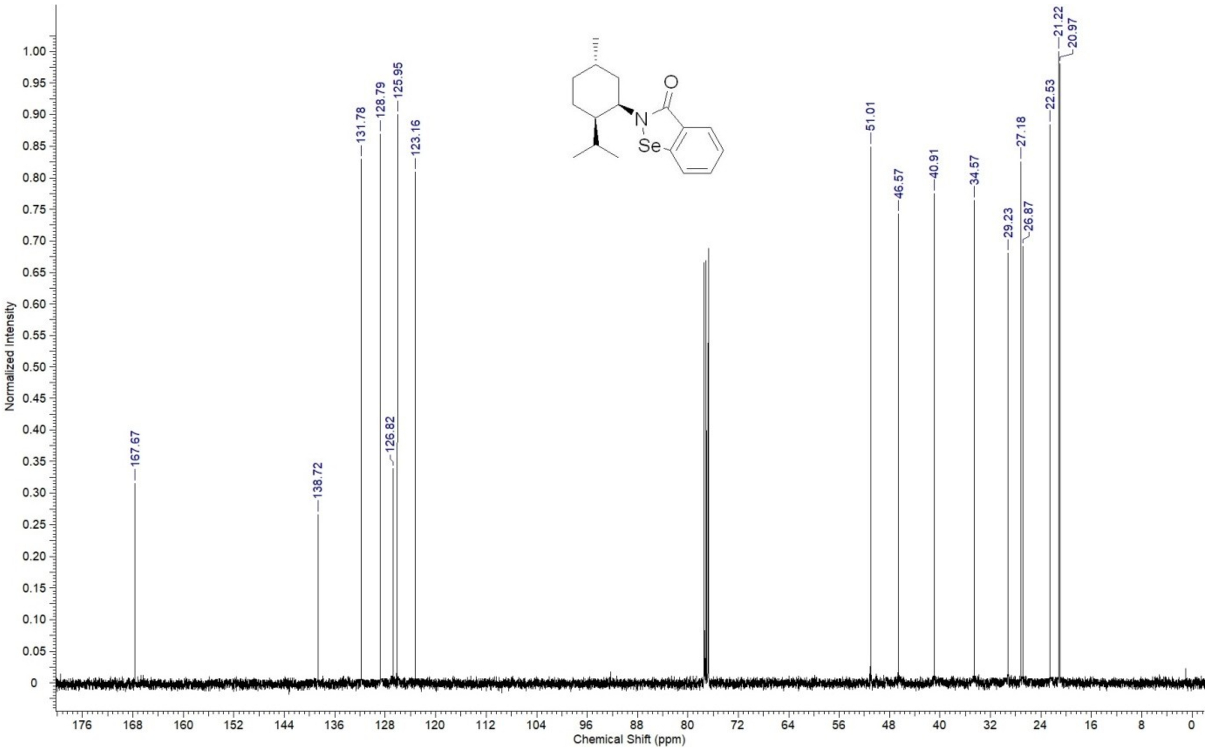

Normalized Intensity
Chemical Shift (ppm)
167.67 138.72 131.78 128.79 126.82 125.95 123.16 51.01 46.57 40.91 34.57 29.23 27.18 26.87 22.53 21.22 20.97
N
Se
O

(b)

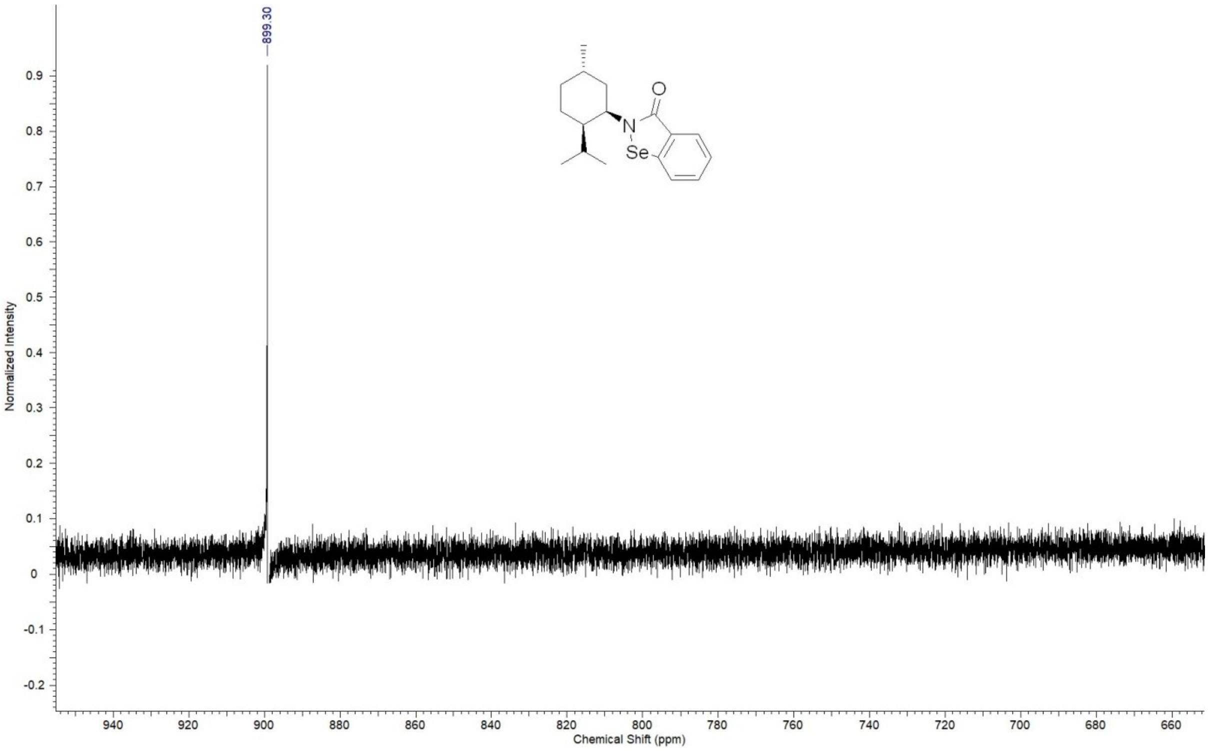

(c)

Figure S2. (a) ^{1}H NMR, (b) ^{13}C NMR, and (c) ^{77}Se NMR spectra of (+)-*N*-(1*R*,2*R*,5*S*)-neomenthyl-1,2-benzisoselenazol-3(2H)-one **23**.

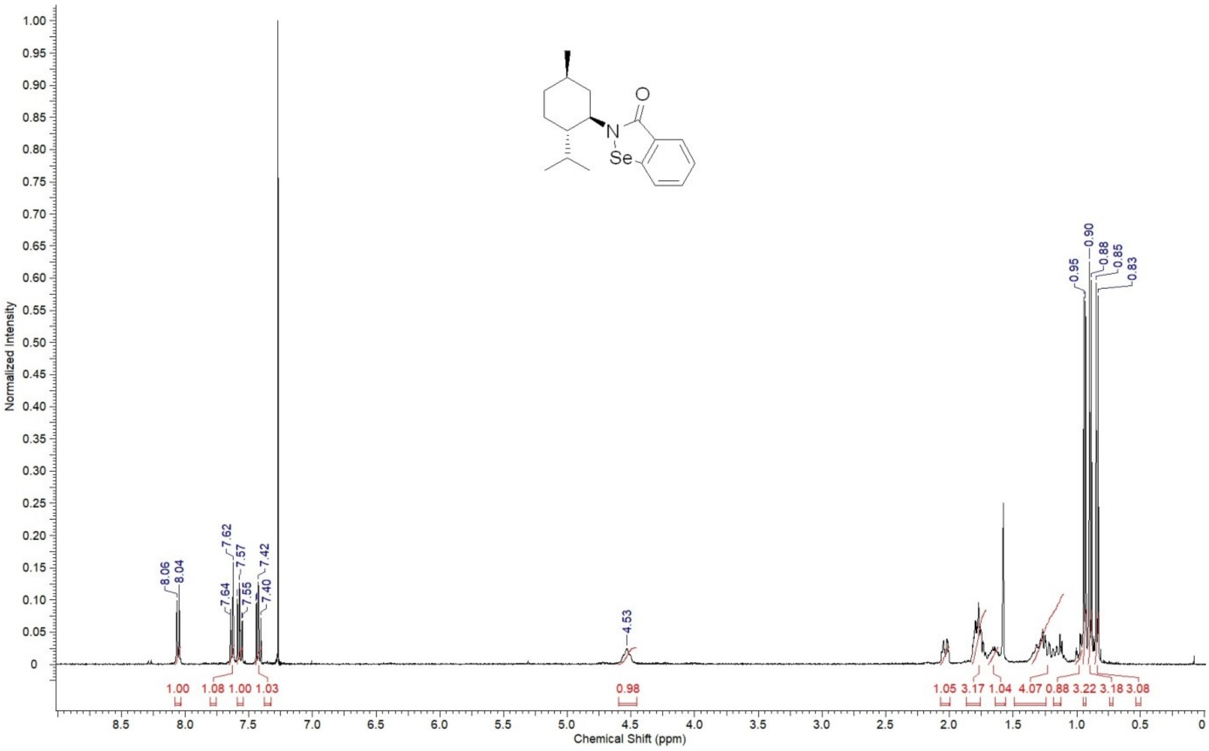

(a)

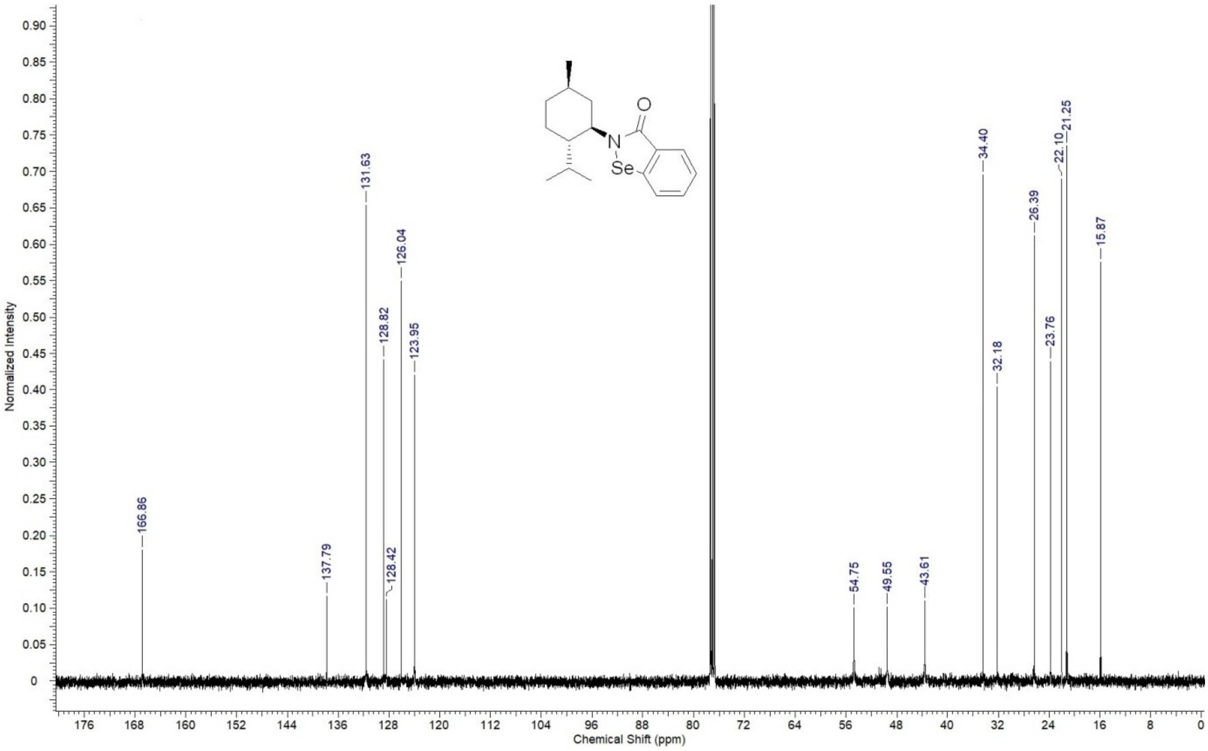

(b)

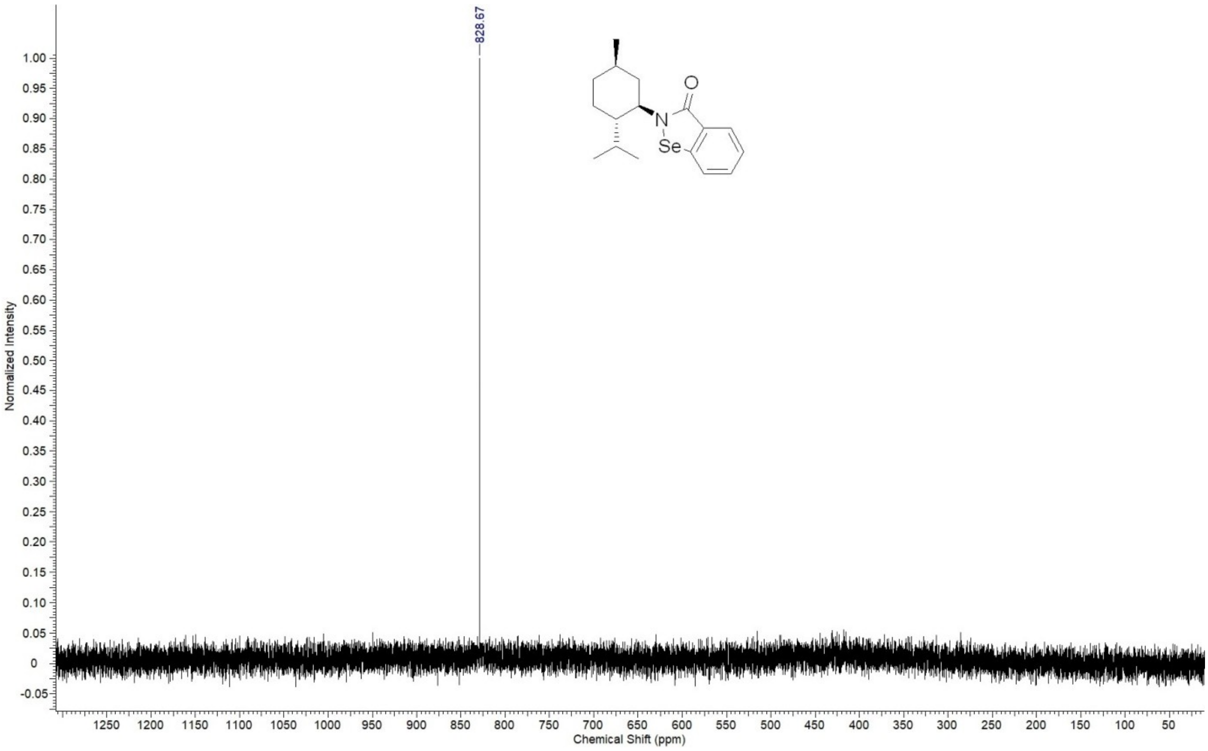

(c)

Figure S3. (a) ^{1}H NMR, (b) ^{13}C NMR, and (c) ^{77}Se NMR spectra of (−)-*N*-(1*R*,2S,5*R*)-menthyl-1,2-benzizoselenazol-3(2H)-one **24**.

materials
MDPI

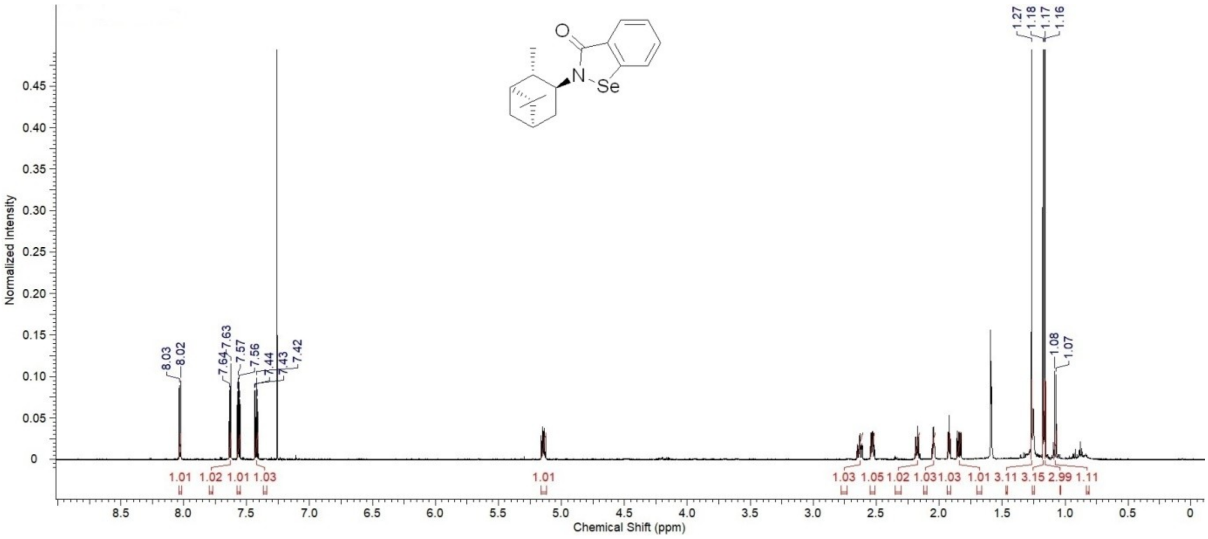

(a)

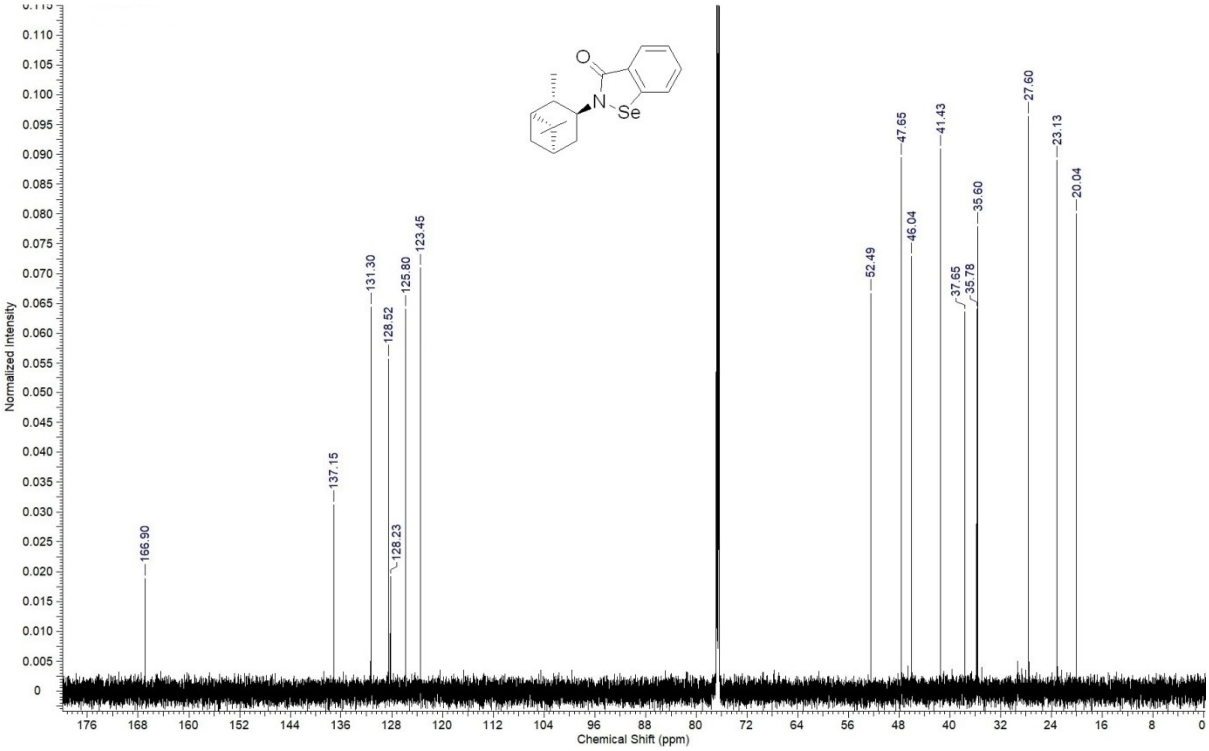

(b)

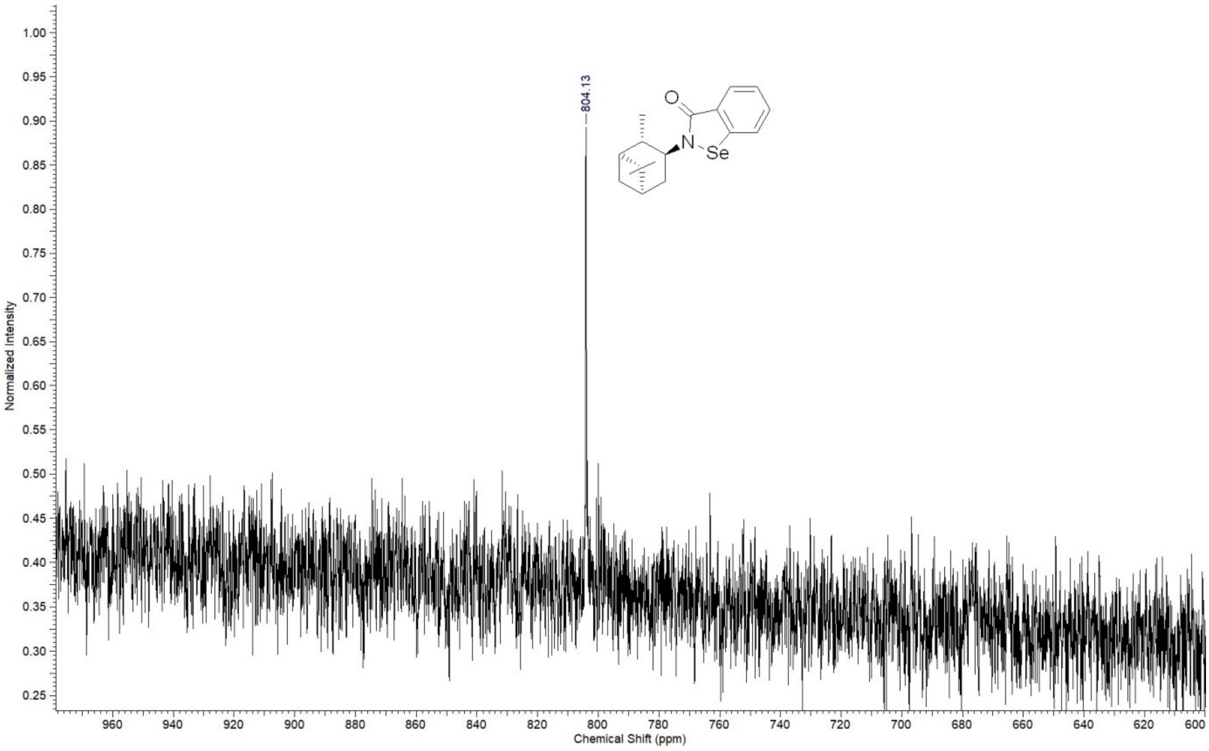

(c)

Figure S4. (a) ^{1}H NMR, (b) ^{13}C NMR, and (c) ^{77}Se NMR spectra (−)-*N*-(1*S*,2S,3*S*,5*R*)-isopinocampheyl-1,2-benzisoselenazol-3(2H)-one **25**.

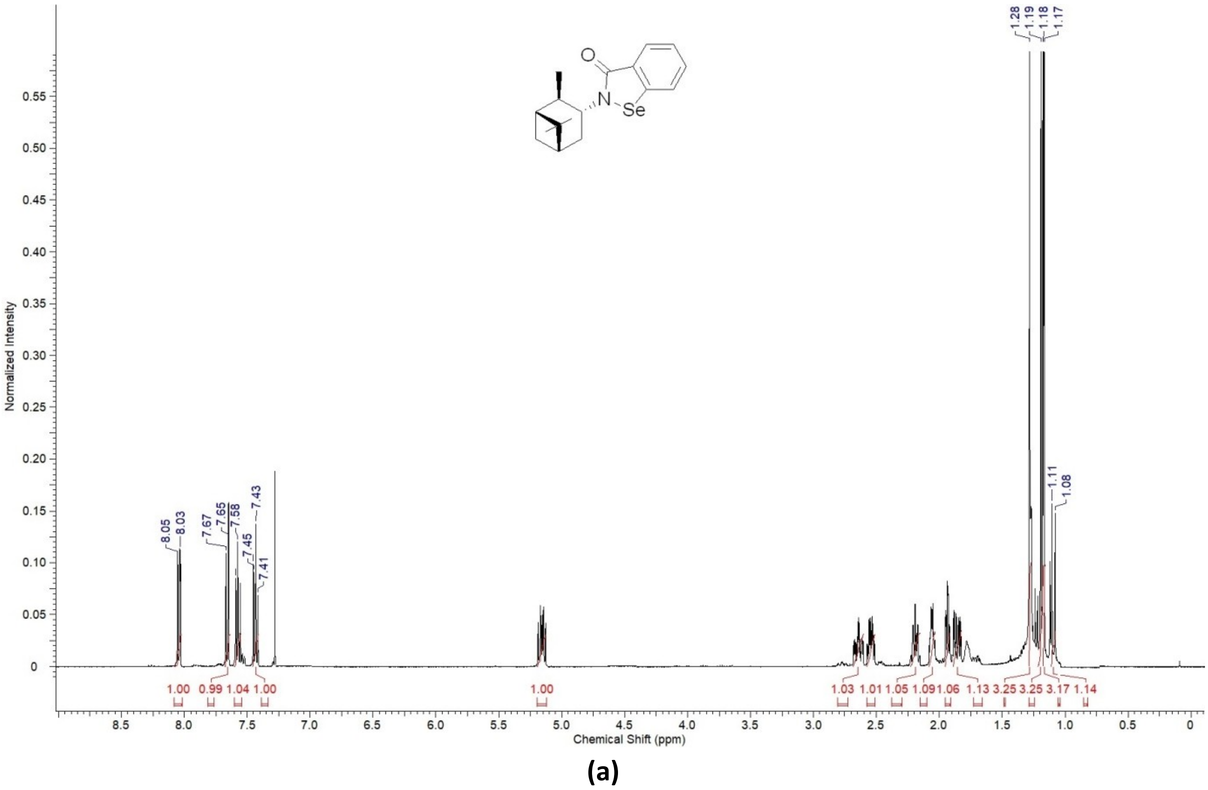

(a)

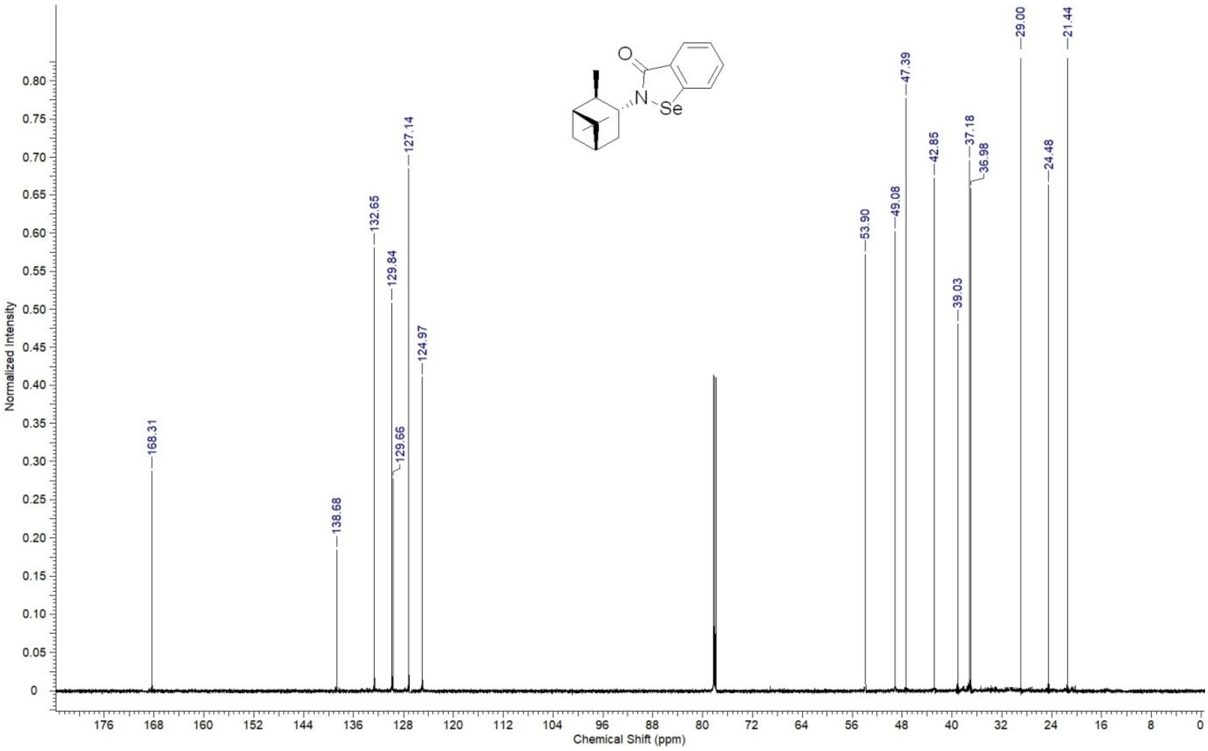

(b)

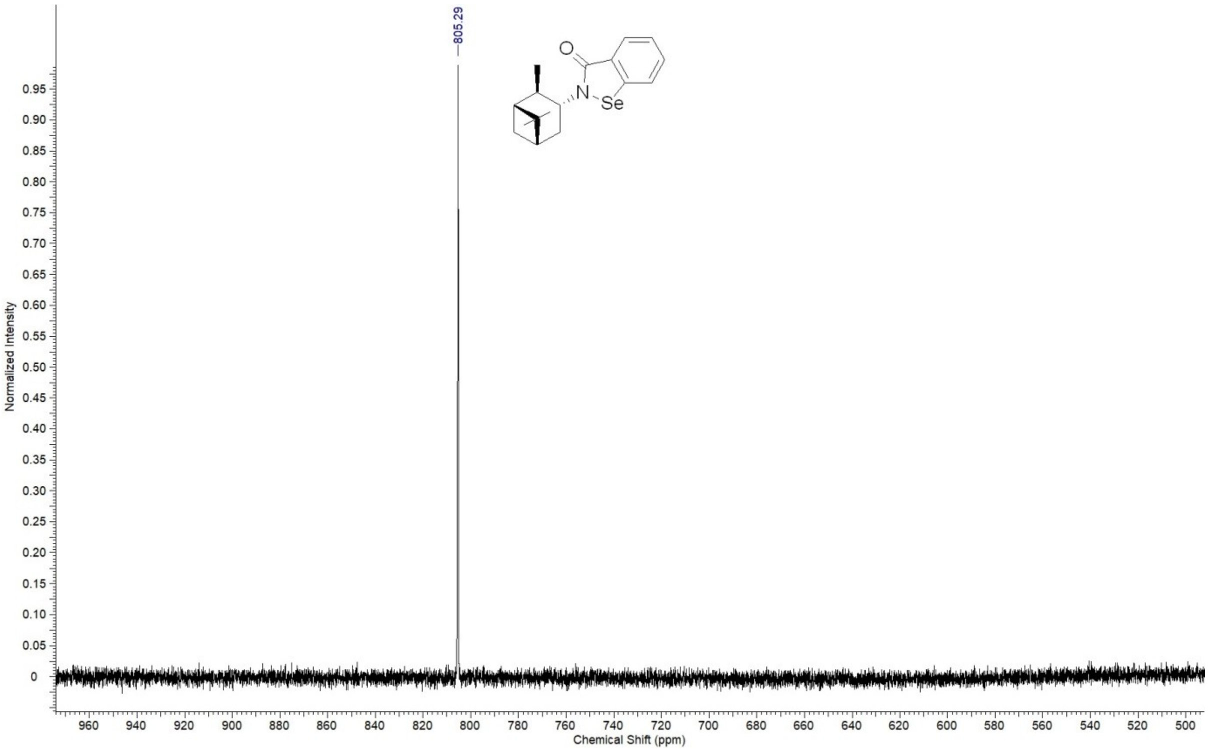

(c)

Figure S5. (a) ^{1}H NMR, (b) ^{13}C NMR, and (c) ^{77}Se NMR spectra (+)-*N*-(1*R*,2*R*,3*R*,5*S*)-isopinocampheyl-1,2-benzisoselenazol-3(2H)-one **26**.

materials

MDPI

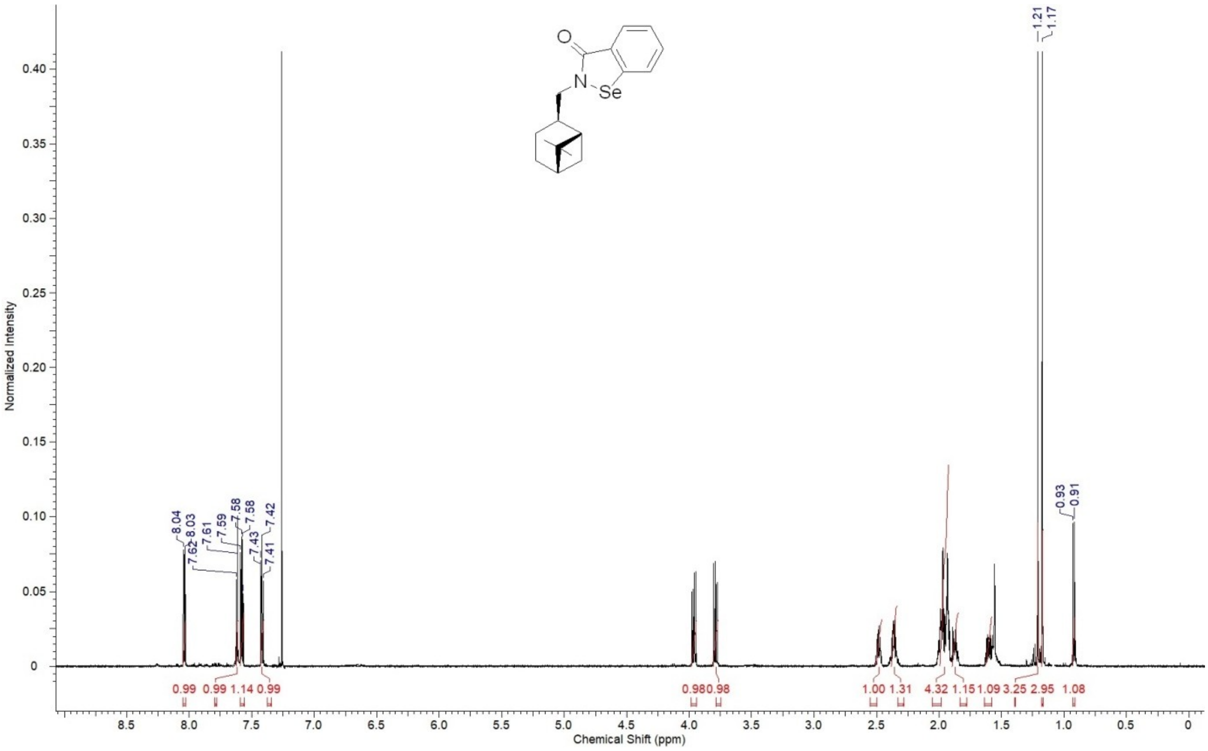

(a)

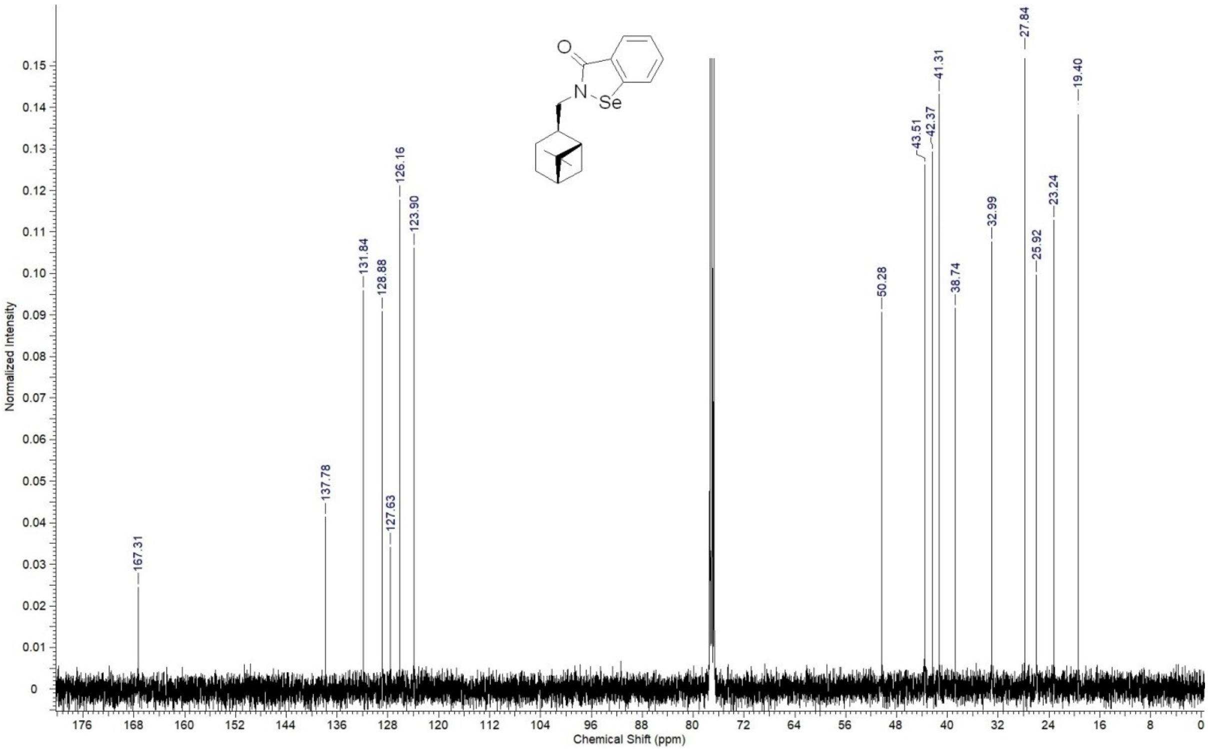

(b)

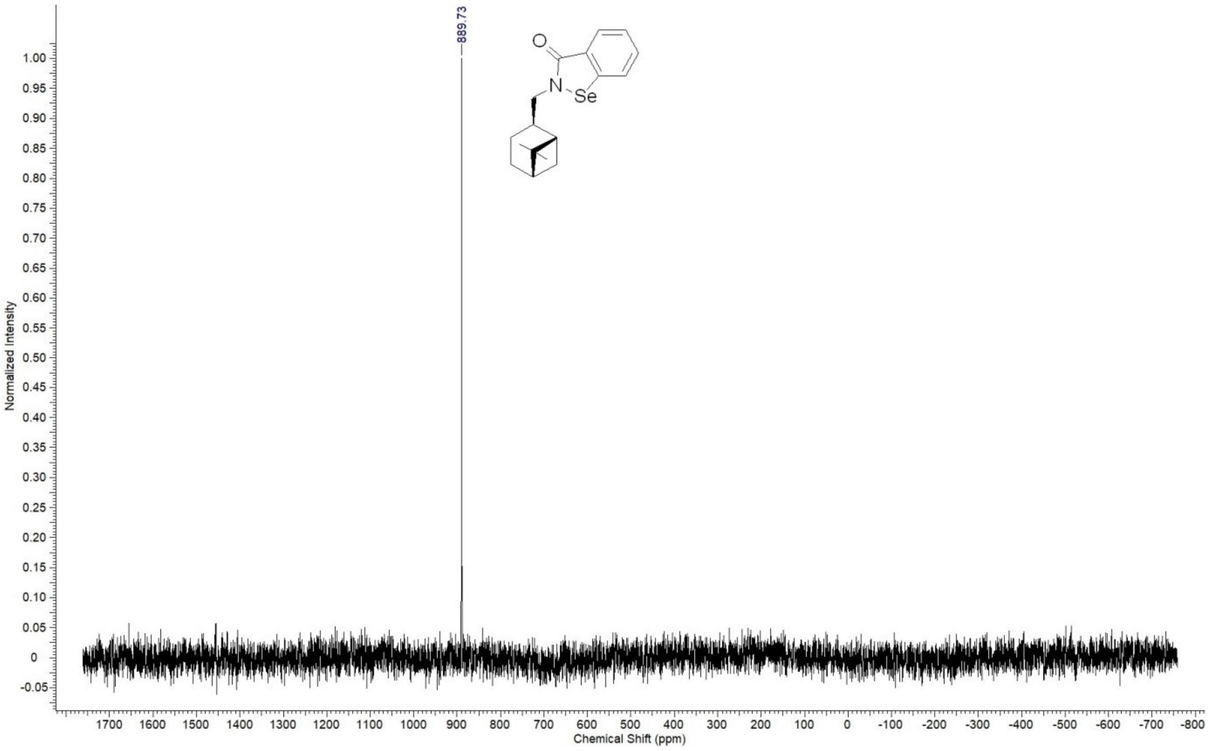

(c)

Figure S6. (a) ^{1}H NMR, (b) ^{13}C NMR, and (c) ^{77}Se NMR spectra of (−)-*N*-(1*S*,2*R*,5*S*)-myrtanyl-1,2-benzisoselenazol-3(2H)-one **27**.
.

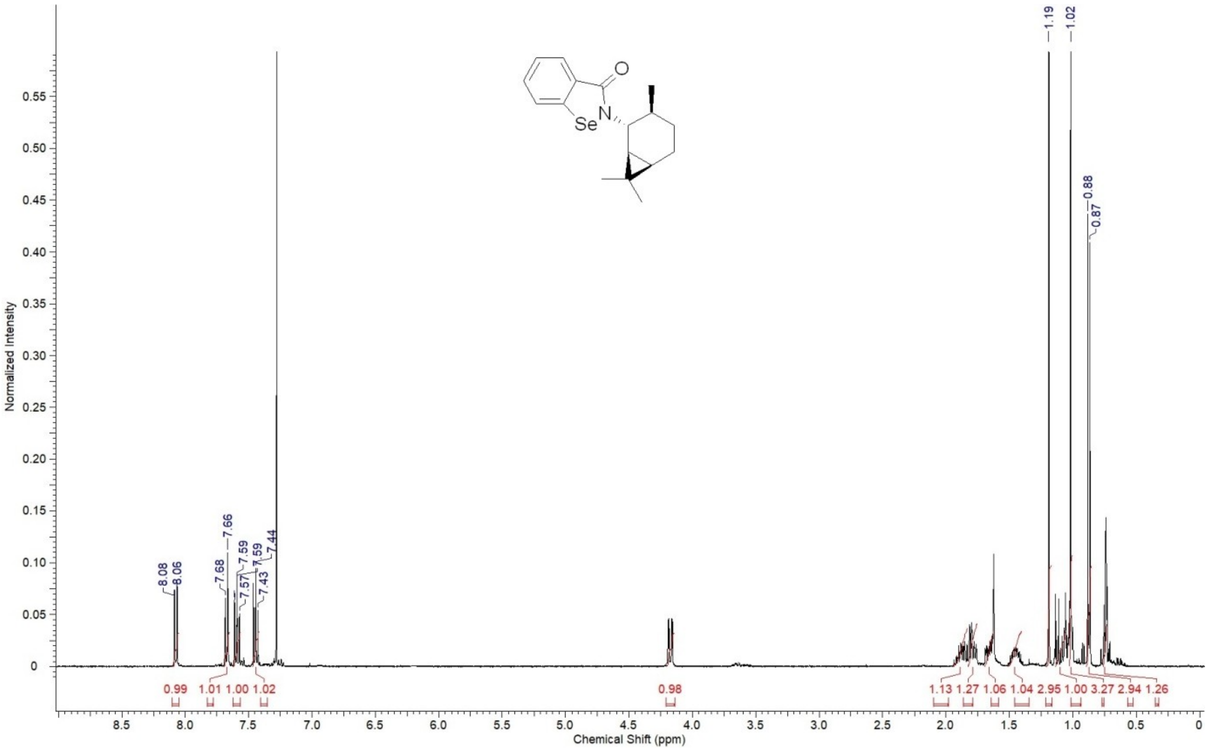

(a)

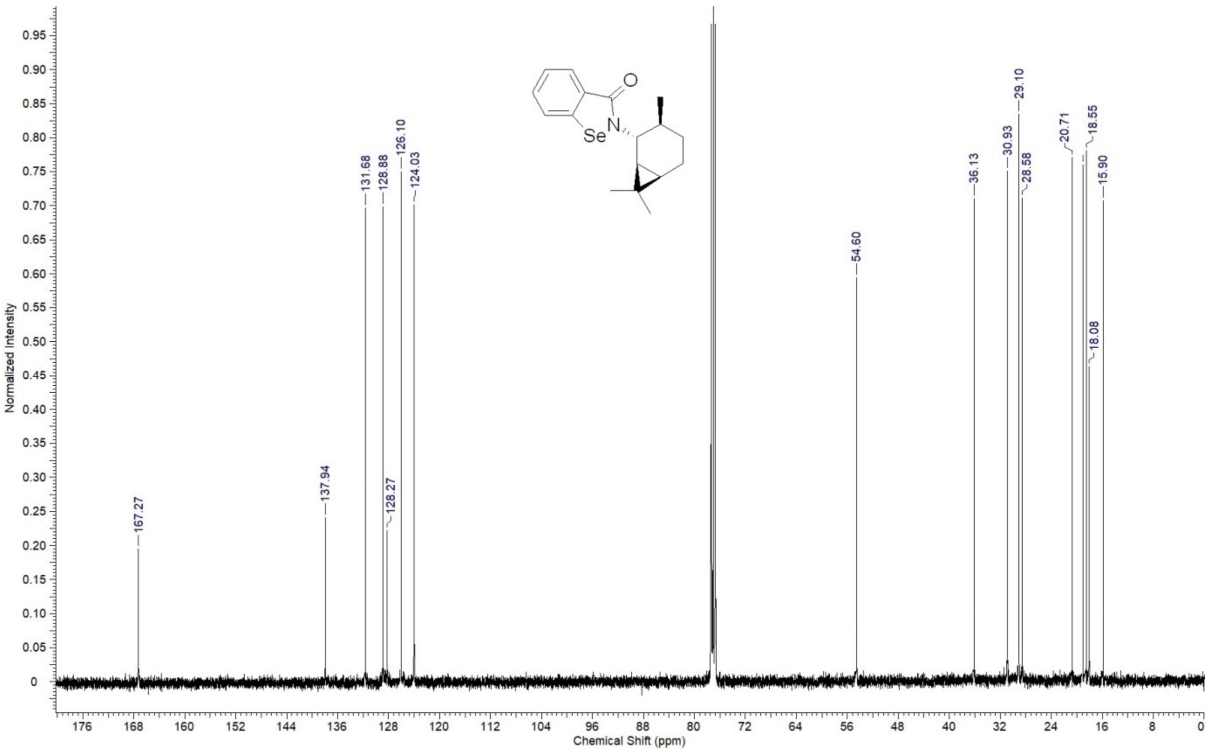

(b)

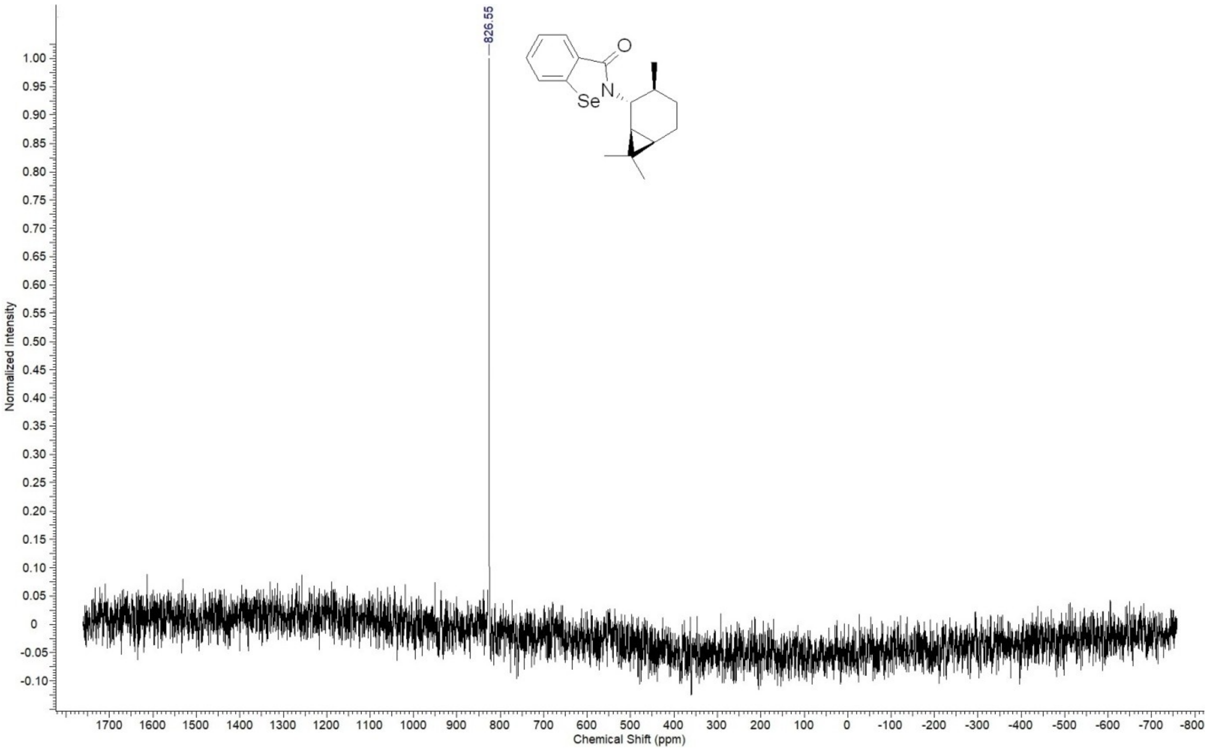

(c)

Figure S7. (a) ^{1}H NMR, (b) ^{13}C NMR, and (c) ^{77}Se NMR spectra of (−)-*N*-(1*S*,2*R*,3*S*,6*R*)-(2-isocaranyl)-1,2-benzisoselenazol-3(2H)-one **28**.

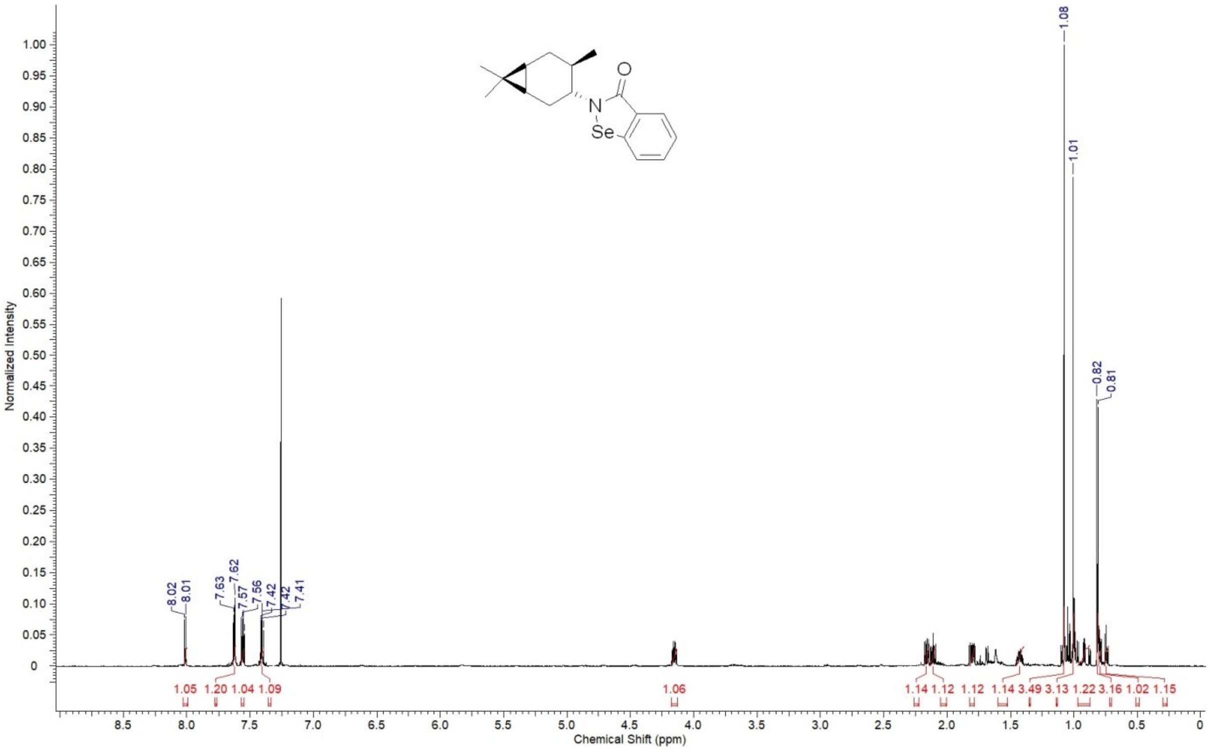

N
Se
O
Normalized Intensity
Chemical Shift (ppm)
1.08
1.01
0.82
0.81
8.02
8.01
7.63
7.62
7.57
7.56
7.42
7.42
7.41
1.05 1.20 1.04 1.09
1.06
1.14 1.12 1.12 1.14 3.49 3.13 1.22 3.16 1.02 1.15

(a)

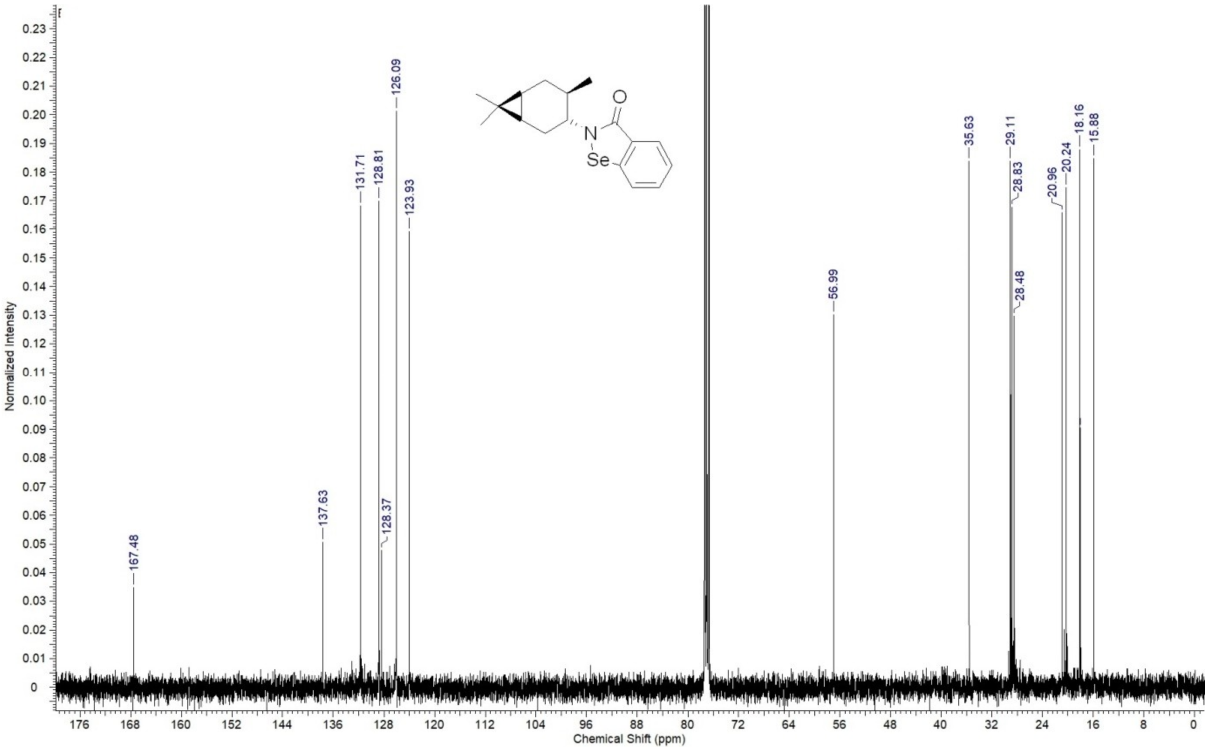

N
Se
O
Normalized Intensity
Chemical Shift (ppm)
167.48
137.63
131.71
128.81
128.37
126.09
123.93
56.99
35.63
29.11
28.83
28.48
20.96
20.24
18.16
15.88

(b)

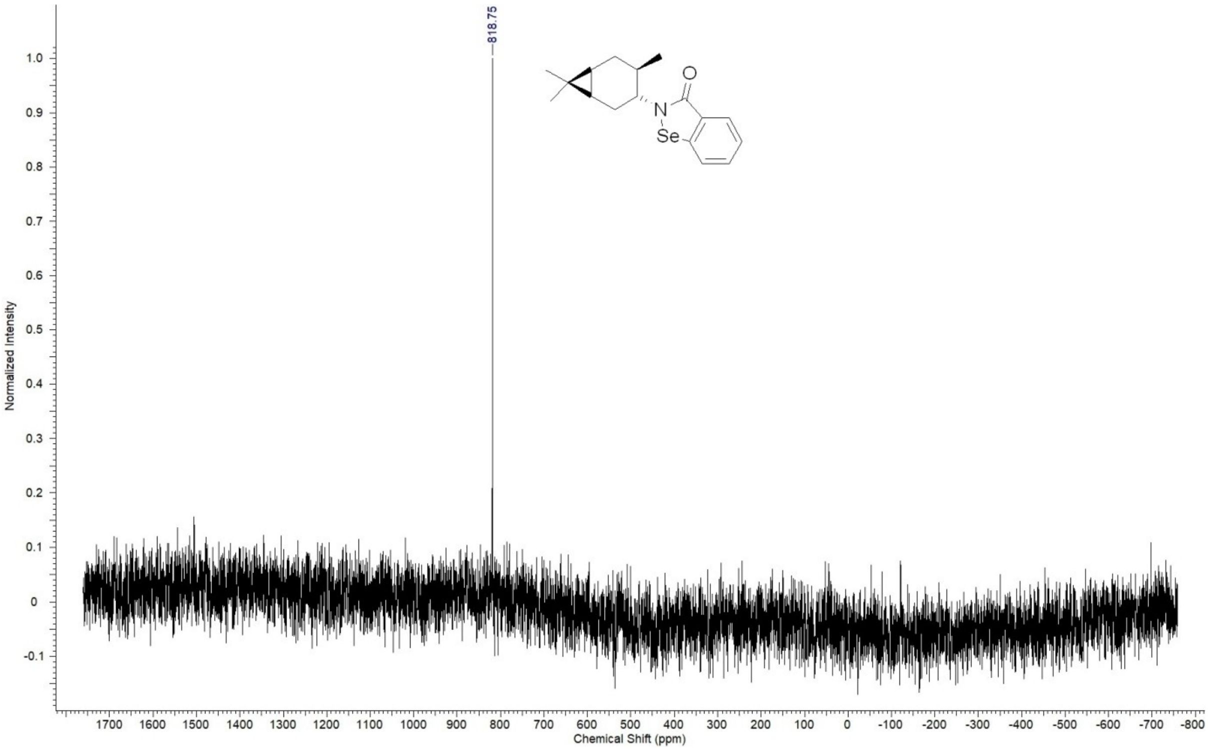

(c)

Figure S8. (a) ^{1}H NMR, (b) ^{13}C NMR, and (c) ^{77}Se NMR −)-*N*-(1*S*,3*R*,4*R*,6*R*)-(4-isocaranyl)-1,2-benzisoselenazol-3(2H)-one **29**.

© 2019 by the authors. Licensee MDPI, Basel, Switzerland. This article is an open access article distributed under the terms and conditions of the Creative Commons Attribution (CC BY) license (http://creativecommons.org/licenses/by/4.0/).
